# The Multimodal Nanomaterials of Phototherapy for Tumor Treatment

**DOI:** 10.14336/AD.2025.0448

**Published:** 2026-05-27

**Authors:** Yanan Bao, Wenxin Chou, Yusen Hou, Shanlin Yang, Yi Li, Yuxia Zhao, Hongyou Zhao

**Affiliations:** ^1^School of Medical Technology, Beijing Institute of Technology, Beijing, 100081, China.; ^2^Department of Burn Plastic Surgery and Wound Repair, Beijing Fengtai You'anmen Hospital Beijing, 100069, China.; ^3^Department of Laser Medicine, the First Medical Centre, Chinese PLA General Hospital, Beijing, 100853, China.; ^4^Department of Oncology, 920th Hospital of Joint Logistics Support Force, Kunming, 650032, China.; ^5^Key Laboratory of Photochemical Conversion and Optoelectronic Materials Technical Institute of Physics and Chemistry Chinese Academy of Sciences, Beijing, 100190, China

**Keywords:** Multimodal collaboration, Photodynamic therapy, Tumor treatment

## Abstract

Tumor is a multiple disease in aged people, which seriously threat their physical and mental health. Photodynamic therapy (PDT) as a novel tumor treatment method has been applied in clinic. However, the limited penetration depth of the excitation light and the hypoxic environment of the tumor limit its therapeutic effect. To breakthrough these two bottlenecks, multimodal nanomaterials that combined two or three therapeutic modalities into a single system were designed, which can significantly improve the effectiveness of tumor treatment. This review firstly summarized the nanomaterials that can mediate the combinations between PDT and other strategies, including photothermal therapy (PTT), chemodynamic therapy (CDT), gas therapy (GT), starvation therapy (ST), photoacidity therapy (PAT), and even the synergistic combination of PDT, PTT, and CDT. Furthermore, the advantages and disadvantages of these combination therapies applied in tumor treatment were compared. Eventually, the challenges of these nanomaterials for clinical applications are discussed.

## 1. Introduction

Cancer, also known as malignant tumor, exhibits an incidence rate closely related to age and is often regarded as a geriatric disease. With the intensification of population aging, the global incidence of cancer has been increasing [1]. Statistics show that the majority of cancer cases occur in individuals aged 60 and above, which is closely associated with factors such as cellular senescence, the accumulation of genetic mutations, and the decline in immune system function. Despite significant advances in modern medicine for cancer treatment, the current approaches still present certain limitations. Chemotherapy is a method used to treat cancer, and during the process, the side effects, effectiveness, and dosage of anti-cancer drugs are important research areas related to global health [2,3]. Surgery may fail to completely remove microscopic lesions [4]. Therefore, exploring more precise, efficient, and less toxic treatment modalities has become a critical direction in medical research. PDT, as an emerging treatment modality, has garnered increasing attention due to its non-invasiveness, good targeting ability, and minimal side effects.

PDT is a treatment method that utilizes photosensitizers to generate reactive oxygen species (ROS) under irradiation to destroy tumor cells. The photosensitizer that was injected into the patient or applied locally will be excited under a specific wavelength of light, transitioning from the ground state to the excited state. Photosensitizers in an excited state can generate ROS in two main ways: Type I mechanism: Photosensitizers in excited states interact directly with substrates in the surrounding environment (such as biomolecules), transferring energy to them, or through an electron transfer process to form free radicals (such as the superoxide anion radical O2-·), which further react to form other types of ROS, such as hydrogen peroxide H_2_O_2_ and the hydroxyl radical OH·. Type II mechanism: More commonly, excited photosensitizers transfer energy directly to nearby oxygen molecules, producing singlet oxygen (^1^O_2_). Singlet oxygen is a very active ROS, which can react with a variety of biomolecules and cause structural damage to cells. ROS can directly attack key biomolecules in cancer cells, including lipids, proteins, and DNA, resulting in cell membrane destruction, enzyme inactivation, and genetic material damage, thereby inducing apoptosis or necrosis of cancer cells [5]. The oxidation potential plays a vital role in the action of ROSs. The standard reduction potential of the superoxide anion is approximately -0.33 V relative to the standard hydrogen electrode (SHE) at pH 7. The hydroxyl radical is an extremely reactive oxidizing agent, with a standard reduction potential estimated between +2.3V and +2.8V (relative to SHE), which indicates that it has a very strong oxidation capacity [6,7]. Oxidation potential of these species, especially for superoxide, depends on the environmental pH [8]. Due to its excellent targeting ability, non-invasiveness, minimal side effects, and capacity for precise targeting of tumor cells, PDT has been widely applied in cancer treatment [9,10]. However, PDT still faces several challenges, such as the limited penetration depth of excitation light and the hypoxic tumor microenvironment, which restrict its therapeutic efficacy. To overcome these limitations, the novel nanoplatforms or compounds that combine two or three therapeutic modalities into a single system were designed. These phototherapy systems can mediate combinations such as PDT with PTT, PDT with CDT, PDT with GT, PDT with ST, PDT with PAT, and even the synergistic combination of PDT, PTT, and CDT. These multimodal materials provide new insights into the diversity and precision of cancer treatment, while also advancing the development and application of phototherapy strategies [11].

**Figure 1.**
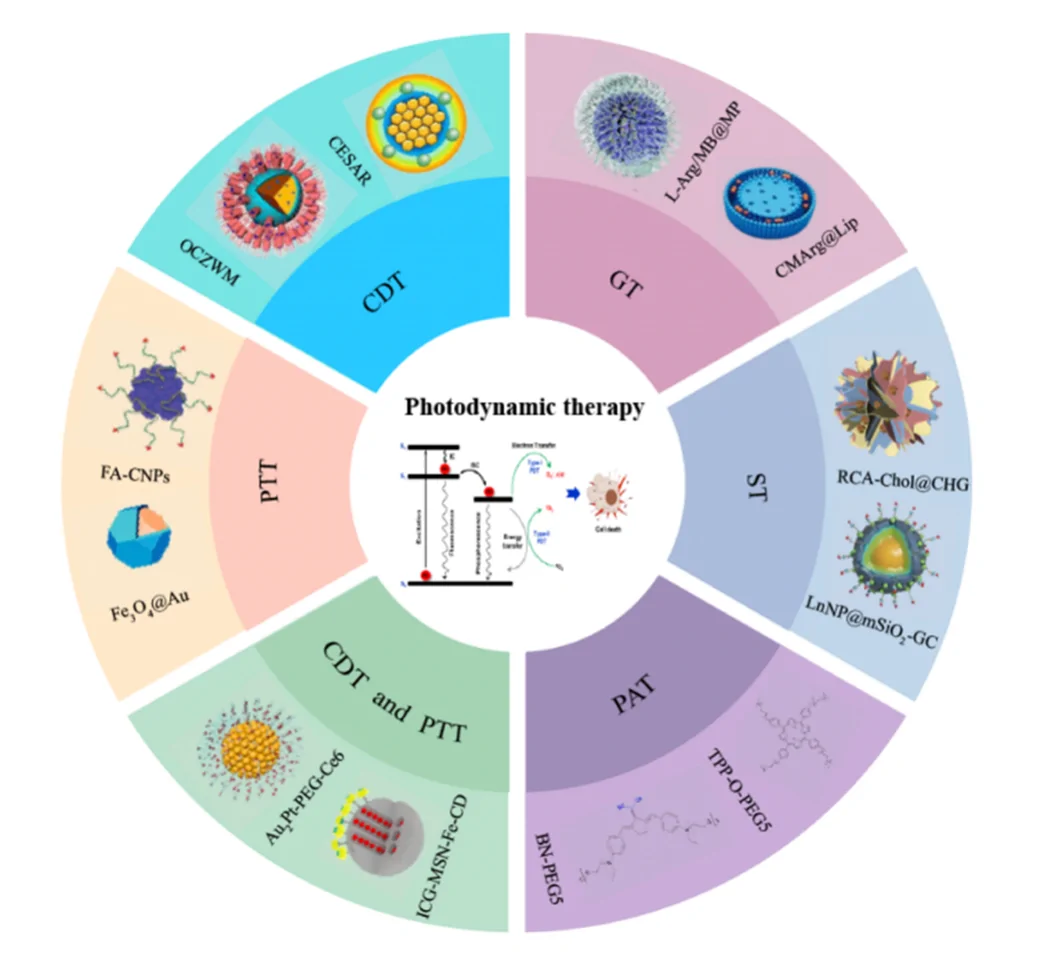
Schematic illustration of the combination of PDT with other therapeutic strategies.

Currently, nanomaterials are widely used in electrochemistry [12], drug delivery [13], photocatalysis [14], cancer treatment [15] and antibiotic detection [16]. This review summarizes the recent advancements in the applications of multimodal photosensitive materials in combination therapies, analyzing the synergistic effects of different therapeutic modalities and their prospects in cancer treatment (Fig. 1). Additionally, this review discusses the current challenges and future directions of multimodal photosensitive materials in applications, with a particular focus on research progress in material design, therapeutic targeting, and the durability of treatment efficacy. Through these comprehensive analyses, this review intends to offer new insights and directions for further research of multimodal photosensitive materials in precision cancer therapy.

## 2. Dual modal photosensitive materials mediate PDT and PTT

PDT and PTT are two distinct tumor treatment modalities. PDT primarily eradicates cancer cells by generating ROS through the activation of photosensitizers under light irradiation. In contrast, PTT utilizes photothermal materials to absorb light energy and convert it into heat, thereby destroying tumor tissues. A key feature of PTT is its ability to directly damage cancer cells by inducing localized heating, creating a high-temperature environment in the tumor region that is inhospitable to cancer cell survival. Since the wavelength of excitation light applied for PTT is typically longer than that for PDT, which is able to more effectively target deep-seated or larger tumors. Importantly, PTT does not rely on oxygen, which addresses the limitations of PDT, such as its restricted treatment depth and susceptibility to the hypoxic tumor microenvironment. In addition, the local heating caused by PTT can improve blood circulation, thus promoting better penetration of photosensitizer into tumor tissue, further enhancing the efficacy of PDT. Therefore, the combination of PDT and PTT leverages the strengths of both modalities, significantly enhancing the therapeutic effect against tumors [17]. The dual modal photosensitive materials mediate PDT and PTT are summarized in Table 1.

**Table 1.** Dual-mode photosensitive materials mediating the synergistic treatment of PDT and PTT.

	PSs	Photothermic agent	Materials	Ref.
**1**	Porphyrins	Metals and metal compound	AuNP–mTHPC, PpIX-GNR-MBA-FA, Collagen-gold hybrid hydrogels	[18–26]
		Organic dyes	PpIX-IR-820@Lipo	
		Other	PDA-PEG-TPPS3, NP-AAG, Porphysome, bTiO2-HA-p, Pp18-lipos	
**2**	Dihydroporphyphenols	Metals and metal compound	NC-Ce6-GNR, Ce6-CuS-TSL, Ce6-PEG-AuNR, CuS-Ce6, GNP-Ce6, CCeT NPs	[27–41]
		Carbon-based	ACEC, HA-Ce6-MnO2@SWNHs, Gd-Ce6@SWNHs, Ce6-RCDs	
		Organic dyes	NP(IR780 + Ce6)	
		Polymer	PDA-Ce6, PPy@BSA-Ce6 NPs, LPP/Ce6	
		Other	PMRCR NPs	
**3**	Metal complex	Metals and metal compound	Fe3O4@Au NCs, Au 25(Capt)18, Ru-GNR-HSANPs, FeNC@PEG, CuS-PEG	[42–49]
		Carbon-based	rGO-Ru-PEG	
		Polymer	Cu(II)/LRu/PDA	
		Other	PEG-NSs + Pd[DMBil1]-PEG750	
**4**	Cyanine dye	Metals and metal compound	Au/MoS2-ICG	[50–57]
		Carbon-based	ICG-HANP/SWCNT(IHANPT)	
		Polymer	ICG-Ag@PANI	
		Organic dyes	HSA-ICG NPs, CaO2/ICG@ZIF-8, SP3NPs, HSA-IR780, IRP/O2	
**5**	Other Organic dyes	Metals and metal compound	CTO-RB-Au, hm-SiO2(AlC4Pc)@Pd	[58–69]
		Carbon-based	CIKP-NP, UCNPs-NGO/ZnPc, SWNHs−TSCuPc nanohybrid	
		Polymer	PPy@MnO2-PEG-MB, PDA-ZnPc+ Nps	
		Organic dyes	UCNP@BSA-RB&IR825, ZnPc-SPC, SiNc-NP	
		Other	WS2@BSA-MB, ZnPc-PA micelles	
**6**	Organic frame	Metals and metal compound	Au@MOF core–shell hybrids, CuS@COF	[86–88]
		Organic dyes	UCNPs@ZrMOF@ICG	
**7**	Plant-based	Metals and metal compound	Fe3O4/SiO2-CUR, Gold nanoshells conjugated with anti-EGFR antibodies, Au NRs/Cur/UCNPs@PBE	[91–96]
		Carbon-based	PC@BPQDs, MWNT–CS–PC	
		Polymer	MnPc@P	
**8**	Others		PHPD-NPs, USFP, PPy@BSA-Astx, PNDI-2T NPs, BT&GA@CL, Cy5.5-BSA-MoS2, F127@CNs-CuS/MnO2, Intermolecular system D - π…π' – A, α-TPE-bisBODIPYs, BP, BP-CuS-FA	[98–108]

### 2.1 Materials Based on Porphyrin Photosensitizers

Porphyrin-based compounds have been widely used as photosensitizers for PDT in combination with PTT. In 2017, Sun *et al.* proposed a strategy using collagen-gold hybrid hydrogels loaded with photosensitizer to achieve synergistic PDT and PTT, demonstrating the system's excellent efficacy in inhibiting tumor growth [18]. In 2021, Tang *et al.* developed a nanoplatform called PpIX-GNR-MBA-FA based on protoporphyrin IX (PpIX). This system incorporated gold nanorods as photothermal agents and enhanced tumor cell internalization and intracellular distribution via folate receptor-mediated targeting, achieving highly efficient PTT/PDT combination cancer therapy [19]. In 2022, Yan *et al.* designed PpIX-IR-820@Lipo nanoparticles by encapsulating PpIX and IR-820 within liposomes. This approach not only improves water solubility and stability, but also enables effective photodynamic/photothermal combination therapy for cervical cancer [20].

mTHPC, a second-generation porphyrin photo-sensitizer, has attracted attention due to its stronger photosensitizing activity and higher singlet oxygen quantum yield. In 2022, Eli Varon *et al.* synthesized AuNP-mTHPC nanocomposites, demonstrating their ability to generate large amounts of ROS upon activation with a 650-nm laser, as well as efficient photothermal conversion when excited by a 532-nm laser. This innovation provides a novel method for treating solid malignant tumors [21]. Additionally, water-soluble metalloporphyrin derivatives such as tetra(p-sulfonatophenyl) porphine (TPPS3) have been extensively applied in PDT and PTT. In 2021, Zmerli *et al.* developed polyethylene glycol (PEG)-functionalized polydopamine nanoparticles (PDA-PEG-TPPS3) with ROS-responsive linkers, enabling spatiotemporal photosensitizer (PS) release and photodynamic activity [22].

**Figure 2.**
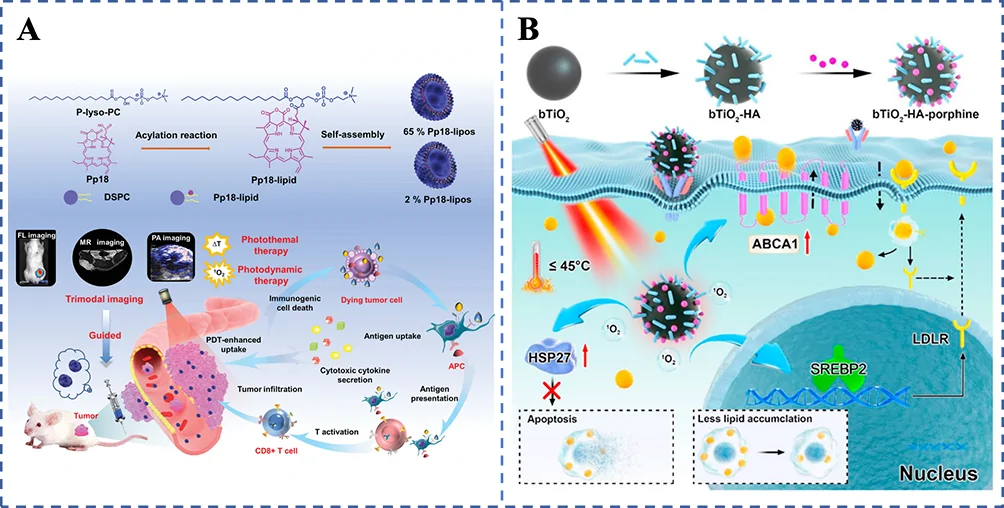
**The application of dual-mode materials Pp18-lipos and bTiO2-based nanoprobes mediating PDT and PTT in cancer treatment. (A)** Illustrative diagram of the preparation process and usage scenarios for Pp18-lipos [25]. Copyright 2020, published by Wiley-VCH Verlag GmbH & Co. KGaA, Weinheim. **(B)** A diagram showing how lipid metabolism occurs in foam cells following phototherapy using bTiO2-based nanoprobes that are activated by an 808 nm NIR laser [26]. Copyright 2022, published by Elsevier B.V.

In 2014, Jin *et al.* revealed the unique structure of porphysomes, which enables a closed-loop transition from PDT to PTT, providing a structure-dependent therapeutic mechanism for cancer treatment [23]. In 2016, Lin and his colleagues developed a novel multifunctional nanoplatform, NP-AAG, by combining a nanoporphyrin-based drug delivery system with a new formulation of heat shock protein 90 (Hsp90) inhibitors for combination therapy in prostate cancer [24]. Through a series of experiments, it has been shown that this nanoplatform can be effectively internalized by prostate cancer cells and show synergistic anti-tumor effects. It enables imaging-guided targeted drug delivery with no significant systemic toxicity, offering promising improvements in the treatment of prostate cancer patients. In 2020, Sun *et al.* designed nanoscale porphyrins composed of Pp18-lipids [25]. By modulating the Pp18-lipid ratio, they achieved fluorescence imaging, photoacoustic imaging, magnetic resonance imaging, and highly efficient synergistic PDT/PTT (Fig. 2A). These studies demonstrated that porphyrin-based photosensitizers hold great potential in constructing multifunctional nanoplatforms, not only enhancing tumor targeting but also enabling multimodal imaging-guided therapies, providing new directions and hope for future cancer treatment. Additionally, Dai *et al.* explored mild phototherapy mediated by black TiO2 nanoprobes in 2022. By loading porphyrins onto hyaluronic acid (HA)-modified black TiO2 nanoparticles, they achieved a reduction in intracellular lipid levels in atherosclerotic foam cells without inducing apoptosis (Fig. 2B) [26].

### 2.2 Materials Based on Chlorin e6 (Ce6) Photosensitizers

In recent years, Ce6, as a typical photosensitizer, has attracted extensive attention due to its strong absorption properties in the long-wave visible region. Composite materials have been developed by combining Ce6 with metal nanoparticles such as gold nanorods (GNRs) and copper chalcogenide nanoparticles as photothermal agents. These materials not only enable efficient PTT, but also enhance the effect of PDT, providing novel and effective strategies for cancer treatment.

**Figure 3.**
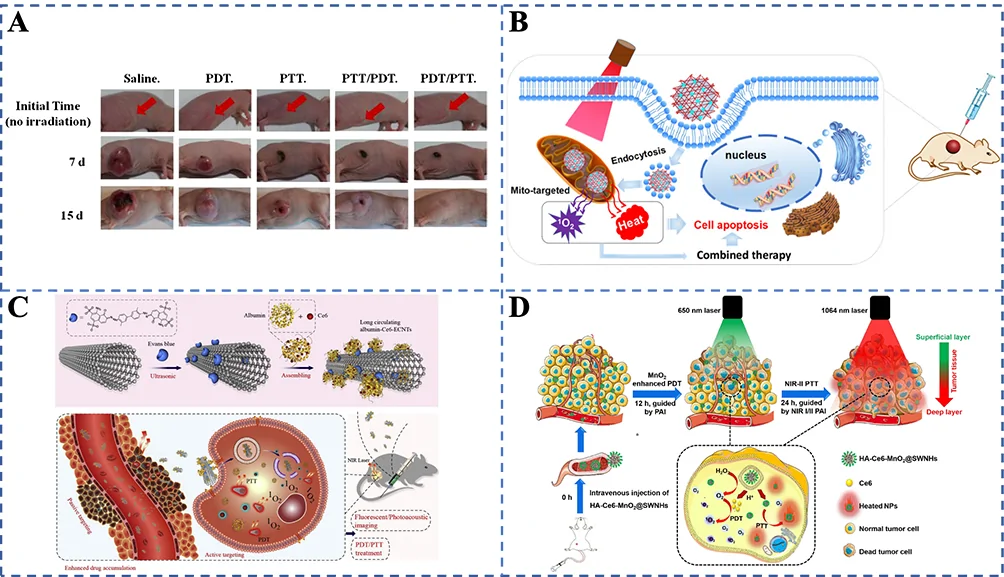
**The application of dual-mode materials. (A)** *In vivo* NIR fluorescence imaging of nude mice with SCC7 tumors following intravenous administration of photoactive agents [27]. Copyright 2013, published by Elsevier B.V.. **(B)** A schematic diagram depicting the synthesis of CCeT nanoparticles and their *in vitro* and i*n vivo* anticancer applications, focusing on mitochondria-targeted synergistic PDT and PTT under near-infrared (NIR) light exposure [32]. Copyright 2018, published by American Chemical Society. **(C)** Development and utilization of an albumin/Ce6 coated EB/carbon nanotube-based delivery system (ACEC) [36]. Copyright 2016, published by Elsevier Ltd..d) A diagram showing the step-by-step process of MnO2 enhanced PDT followed by NIR-II PTT combination treatment [37]. Copyright 2022, published by Elsevier Ltd..

Kim *et al.* constructed a bifunctional nanosystem, NC-Ce6-GNR in 2013 by loading gold nanorods and Ce6 into chitosan-functionalized Pluronic nanogels. By adjusting the loading method, the system achieved independent functionalities for PDT and PTT [27]. Experiments demonstrated that performing PDT followed by PTT significantly enhanced tumor ablation, proving the synergistic effect of PDT and PTT is sequence-dependent (Figure 3A). Similarly, Zhang *et al.* designed a Ce6-PEG-AuNR nanotherapeutic agent in 2017, which remained stable under normal physiological conditions and released Ce6 in the acidic tumor microenvironment to restore fluorescence for tumor localization. Both *in vitro* and *in vivo* experiments have shown that the combination therapy is more effective than monotherapy [28]. In 2023, Kim *et al*. explored the effects of PDT-PTT under 635 nm laser irradiation using a gold nanoscale double pyramid combined with Ce6. The therapeutic effect was evaluated *in vitro* and *in vivo*. The results revealed that combination therapy increased the temperature by approximately 20°C and reduced cell viability by 12% compared to PDT alone. This approach simultaneously induced apoptosis and necrosis, suppressed tumor recurrence, and compensated for the limitations of traditional therapies [29]. In all of these studies, Ce6 acted as a photosensitizer, while metal-based nanomaterials acted as a photothermal agent to achieve a synergistic effect between PDT and PTT.

In 2016, Tan *et al*. developed folate-coupled heat sensitive liposomes (Ce6-CuS-TSL) that use the heat generated by CuS under near-infrared laser irradiation to trigger the release of Ce6, thus enabling the combination of PTT with PDT [30]. Bharathiraja *et al.* designed CuS-Ce6 nanoparticles (NPs) that could produce singlet oxygen and heat under laser irradiation with different wavelengths, demonstrating their potential in image-guided phototherapy [31]. In 2023, Xiang *et al.* successfully engineered a multifunctional system called CCeT NPs (Figure 3B). Their research confirmed that these nanoparticles could localize to the mitochondria of cancer cells. Under 630 nm laser excitation, CCeT NPs efficiently generated singlet oxygen, while under 808 nm laser irradiation, they exhibited excellent photothermal conversion efficiency. Both *in vitro* and *in vivo* experiments showed that CCeT nanoparticles effectively inhibited tumor growth through the synergistic effect of PDT and PTT, showing higher efficacy than single mode therapy [32]. These studies used nanoparticles based on copper chalcogenide compounds as photothermal agents to achieve synergistic therapeutic effects.

In addition to metal compound nanoparticles, which are commonly used as photothermal agents, polydopamine (PDA) and polypyrrole (PPy) are also excellent photothermal conversion materials. These materials can convert light energy into heat under near-infrared (NIR) irradiation, raise local temperatures to directly kill tumor cells, or enhance the efficacy of PDT by raising local temperatures. In 2015, Zhang's team synthesized Ce6-modified polydopamine nanospheres (PDA-Ce6) [33], and Song’s group developed PPy@BSA-Ce6 nanoparticles in the same year [34]. Both materials demonstrated good biocompatibility and highly efficient dual-mode PDT/PTT effects. In recent years, Wu and colleagues have focused on clay-polymerized polypyrrole nanodisks, which exhibit excellent colloidal stability and biocompatibility, with a loading capacity for Ce6 of up to approximately 89.2%. The study showed that the photothermal conversion efficiency of these nanodisks, known as LPP, reached 45.7% under 980 nm near-infrared laser irradiation, exceeding its performance under 808 nm laser irradiation. Both *in vitro* and *in vivo* experiments have demonstrated that Ce6-loaded LPP nanodisks possess combined photothermal and photodynamic therapeutic effects, significantly inhibiting the malignant proliferation of tumors [35].

Carbon-based materials have been extensively studied to achieve efficient and safe photothermal therapies.Xie *et al*. prepared the ACEC composite (Figure 3C) [36], and Zhang’s team developed HA-Ce6-MnO2@SWNHs (Figure 3D) nanohybrids, both of which demonstrated the ability to prolong circulation time and respond to the tumor microenvironment to alleviate tumor hypoxia.[37] These studies confirmed that the combination of PDT and PTT significantly enhances tumor ablation efficacy with minimal side effects. Yang and colleagues developed single-walled carbon nanohorns loaded with Gd3+ and chlorin e6 (Gd-Ce6@SWNHs), which not only promoted systemic antitumor immune responses but also eliminated distant metastatic lesions, offering a novel approach to combat advanced metastatic cancer [38]. In the same year, Sun and his team proposed a new strategy of loading a small amount of photosensitizer onto photothermal agents, activated by a single near-infrared (NIR) laser. By amide condensation reaction, a small amount of Ce6 (mass ratio 0.56%) was loaded onto amino-rich red-emitting carbon dots (RCDs), forming Ce6-RCDs. *In vitro* and *in vivo*, Ce6-RCDs have demonstrated excellent cancer treatment at low laser power densities (0.50 Wcm-2,671 nm) and it also has multi-modal imaging capabilities, including fluorescence, photoacoustic and photothermal imaging, which can guide the phototherapy process. This provided a strategy to enhance the efficacy of cancer phototherapy [39]. Furthermore, the switchable photodynamic therapy (Switch-PDT) nanoplatform NP (IR780 + Ce6) proposed by Zhang and the multifunctional nanocomposite PMRCR NPs designed by Chen emphasized the importance of combining PDT and PTT for effective tumor ablation and demonstrated how to break through the characteristics of hypoxia in the tumor microenvironment to improve the therapeutic effect [40,41].

### 2.3 Materials Based on Metal Complex Photosensitizers

In recent years, the application of complexes containing metal centers as photosensitizers has gradually gained attention. These metal-based photosensitizers can effectively absorb light and promote electron or energy transfer, demonstrating unique advantages in specific applications.

**Figure 4.**
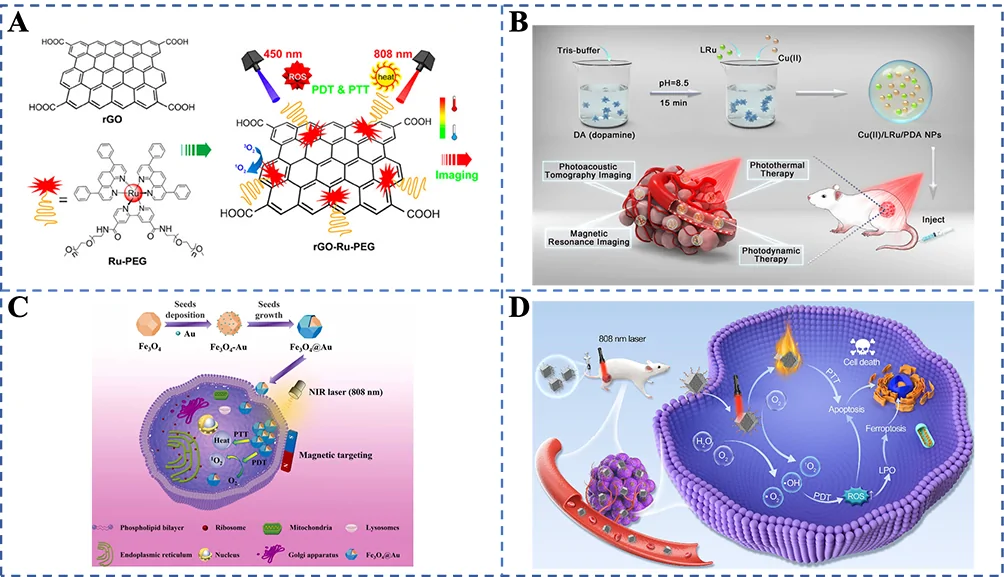
**The application of dual-mode materials rGO-Ru-PEG, Cu(II)/LRu/PDA NPs, Fe3O4@Au NCs and FeNC mediating PDT and PTT in cancer treatment. (A**) A diagram depicting the formation process of rGO-Ru-PEG [42]. Copyright 2017, published by American Chemical Society. **(B)** Illustrative diagram of the preparation and uses of Cu(II)/LRu/PDA nanoparticles for MR/PAT imaging and PDT/PTT dual-mode treatment [45]. Copyright 2022, published by American Chemical Society. **(C)** A diagram illustrating the preparation method of Fe3O4@Au nanocomposites and their use in targeted PTT/PDT synergistic treatment for tumors, activated by 808 nm laser irradiation [47]. Copyright 2023, published by Elsevier B.V.. **(D)** A schematic representation of the *in vivo* combined PDT and PTT effects of FeNC@PEG [49]. Copyright 2024, published by Elsevier B.V..

In 2017, Zhang *et al.* constructed a nanohybrid material consisting of reduced graphene oxide (rGO) supported with pegylated ruthenium (II) complexs (Ru-PEG), named rGO-Ru-PEG (Fig. 4A). This material exhibited excellent pH-sensitive and photothermal-responsive release characteristics and was efficiently taken up by A549 cancer cells, localizing in lysosomes. Experiments showed that this composite material achieved better anticancer effects through combined PTT/PDT compared to single-mode treatments and could be used for *in vivo* photothermal imaging and tumor ablation [42]. In 2019, Pei Liu *et al.* utilized biocompatible gold nanoclusters Au25(Capt)18 for PTT and PDT in cutaneous squamous cell carcinoma (cSCC). The results demonstrated that this material not only inhibited cSCC cell proliferation but also promoted the suppression of tumor growth in mice. Additionally, the anti-tumor immune response induced by Au25(Capt)18 provided new insights into skin cancer treatment [43]. In 2021, Riley *et al.* employed silica-core/gold-shell nanoshells (PEG-NSs) for PTT and palladium-based diimine photosensitizers (Pd[DMBil1]-PEG750) for PDT to evaluate the efficacy of combining PTT and PDT in treating triple-negative breast cancer. The study demonstrated that this combination strategy was superior to single-mode therapies in inducing cell death, primarily promoting apoptotic cell death and reducing adverse effects associated with necrosis [44]. In 2022, Zhang *et al.* designed Cu(II)/LRu/PDA NPs (Fig. 4B), to realize dual-mode PDT/PTT therapy guided by magnetic resonance/photoacoustic tomography [45].In the same year, Shi *et al.* developed a human serum albumin (HSA)-coated gold nanorod (GNR) nanocarrier loaded with ruthenium complexes for synergistic PDT/PTT, further proving its significant synergistic anticancer effects at low concentrations [46]. In 2023, Zhou *et al.* designed and synthesized novel multifunctional Fe3O4@Au nanocomposites (NCs) (Fig. 4C). This material acts as both a photothermal conversion agent and a photosensitizer and can realize the synergistic effect of PTT and PDT under the irradiation of a single wavelength 808 nm low power laser. It shows high photothermal conversion rate and good biocompatibility [47]. In the same year, Li *et al.* focused on the mechanisms of copper sulfide (CuS) nanoparticles for PDT and PTT, demonstrating their anticancer potential both *in vitro* and *in vivo* [48]. In 2024, Li and his team developed iron-nitrogen-carbon (FeNC) nanoparticles (Fig. 4D) with excellent photostability as photosensitizers for combined therapy. Under 808 nm laser irradiation, FeNC nanoparticles generated both Type I and Type II ROS while providing heat, with photothermal conversion efficiency of up to 34%. Experimental validation confirmed the effectiveness of FeNC as a photosensitizer in inhibiting tumors, inducing apoptosis, and promoting ferroptosis in cancer cells [49].

Therefore, metal-based compounds can serve as effective photosensitizers for cancer treatment under specific conditions, particularly when combined with PDT and PTT, demonstrating significant synergistic effects and therapeutic potential.

### 2.4 Materials Based on Cyanine Dye Photosensitizers

**Figure 5.**
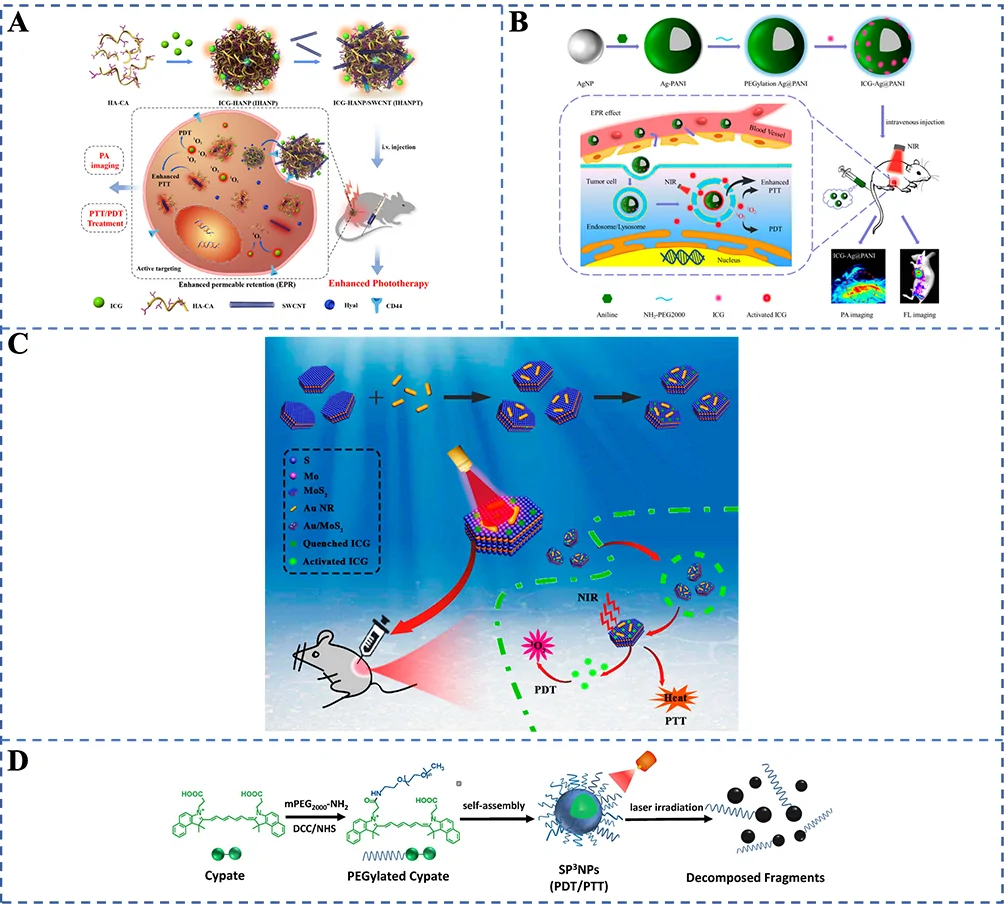
**The application of dual-mode materials IHANPT, ICG-Ag@PANI, AuNRs/MoS2-ICG and SP3NPs mediating PDT and PTT in cancer treatment. (A)** The design and functionality of the dual-targeted phototherapy agent, ICG-linked threadlike nanoparticles (IHANPT) [51]. Copyright 2016, published by American Chemical Society. **(B)** A diagram showing the preparation process and functional mechanism of ICG-Ag@PANI theranostic nanocomposites, utilized for photoacoustic/fluorescence imaging-guided PTT and PDT [52]. Copyright 2016, published by American Chemical Society. **(C)** Diagram illustrating the synthesis method and operational mechanism of the Au/MoS2−ICG nanoplatform, designed to achieve concurrent PDT and synergistic plasmonic PTT under low-power single continuous wave (CW) NIR laser illumination (808 nm, 0.2 W/cm^2^) [53]. Copyright 2019, published by American Chemical Society.d) A diagram showing the synthesis of PEGylated cypate, the formation of its self-assembled nanoparticles (SP3NPs), and the laser-induced decomposition of these nanoparticles [57]. Copyright 2016, published by Ivyspring International.

Cyanine dye-based photosensitizers, such as indocyanine green (ICG), IR-780, and cypate, have demonstrated significant potential in the biomedical field, particularly in cancer optical imaging and PDT, due to their absorption and emission properties in the near-infrared (NIR) region. Although ICG has relatively weak capability of singlet oxygen generation, which can be overcome by combining it with other materials. For example, in 2014, Sheng *et al.* developed a programmed assembly strategy to fabricate human serum albumin (HSA)-indocyanine green (ICG) nanoparticles (HSA-ICG NPs). They enabled clear identification of tumors and tumor margins through dual-modal imaging using near-infrared fluorescence (NIRF) and photoacoustic (PA) techniques [50]. In 2016, Wang and his team developed a nanocomposite called IHANPT (Fig. 5A), which combined ICG with hyaluronic acid nanoparticles (HANPs) and encapsulated single-walled carbon nanotubes (SWCNTs). This nanocomposite exhibited excellent tumor-targeting ability both *in vitro* and *in vivo*, enhanced photothermal conversion efficiency, and effectively induced tumor cell death without significant toxic side effects. This provided a new strategy for photoacoustic imaging-guided photothermal and photodynamic combination therapy in cancer treatment [51]. In the same year, Tan *et al.* constructed polyethylene glycol-coated silver nanoparticle core/polyaniline shell nanocomposites loaded with ICG (ICG-Ag@PANI) (Fig. 5B).

Under a single 808 nm NIR laser irradiation, these nanocomposites achieved efficient PTT/PDT combination therapy, significantly enhancing HeLa cell killing and tumor growth inhibition [52]. Younis *et al.* proposed an AuNRs/MoS2-ICG strategy (Fig. 5C) in 2017, utilizing AuNRs/MoS2 dual plasmonic photothermal nanoagents. This system rapidly increased temperature within a short time and promoted the release of electrostatically bound ICG to induce PDT, enabling simultaneous PDT/PTT [53]. In 2023, Zhang and his team developed an oxidative stress amplifier, CaO2/ICG@ZIF-8, which not only significantly suppressed tumor growth but also exhibited excellent biocompatibility and safety, providing a promising strategy for synergistic PDT/PTT cancer treatment [54].

As a near-infrared fluorescent dye, IR-780 has exhibited significant applicability and promising application potential. In 2014, Jiang *et al.* encapsulated IR-780 in human serum albumin nanoparticles (HSA-IR780 NPs) for combined photothermal and photodynamic cancer therapy. These nanoparticles showed a 1,000-fold increase in solubility and reduced toxicity. Under 808 nm laser irradiation, they generated heat and ROS, inhibiting tumor growth without cytotoxicity in healthy mice, making them a promising candidate for cancer phototherapy [55]. In recent years, Chen *et al.* developed a mitochondria-targeted liquid perfluorocarbon (PFC)-based oxygen delivery system for imaging-guided synergistic PDT/PTT. This system exhibited excellent colloidal stability, high oxygen-loading capacity, superior photothermal and photodynamic performance, and mitochondria-targeting capability [56]. Subsequently, Miao *et al.* designed and fabricated a novel nanodrug, SP3NPs, self-assembled from polyethylene glycolated cypate in 2016. This nanodrug possessed photodegradable, photodynamic, and photothermal properties (Figure 5D). *In vitro* experiment results confirmed that it could generate singlet oxygen and heat under NIR laser irradiation. *In vivo* experiment results showed that SP3NPs accumulated within tumor tissues through the enhanced permeability and retention (EPR) effect, enabling imaging-guided photodynamic and photothermal combination therapy. This approach effectively eradicated tumors and prolonged the survival of mice [57].

### 2.5 Materials Based on Other Organic Dye Photosensitizers

Organic dyes play a crucial role as photosensitizers in the field of phototherapy. Compounds such as phthalocyanines (Pcs) [58–65], rose bengal (RB) [66, 67], and methylene blue [68, 69] exhibit excellent photosensitizing properties and are frequently used to construct multifunctional nanoplatforms for combined PDT and PTT research. The therapeutic effectiveness of the treatment has been substantiated by both *in vitro* and *in vivo* experiments, particularly in the context of tumor suppression.

### 2.6 Materials Based on Organic Framework Photosensitizers

**Figure 6.**
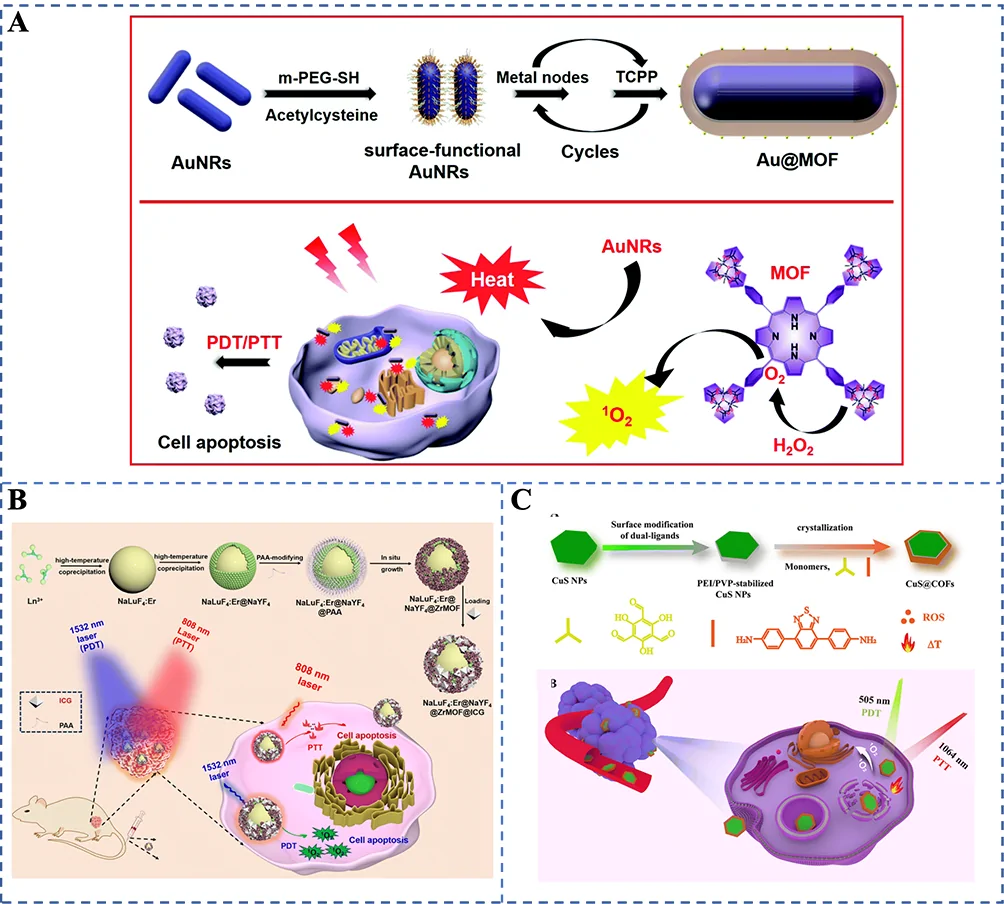
**The application of dual-mode materials Au@MOF, UCNPs@ZrMOF@ICG and CuS@COF mediating PDT and PTT in cancer treatment**. **(A)** The preparation method and operational mechanism of Au@MOF core–shell hybrids [86]. Copyright 2021, published by The Royal Society of Chemistry. **(B)** A diagram illustrating the synthesis process of UCNPs@ZrMOF@ICG nanocomposites and their potential application in antitumor treatments, integrating 1532 nm light-mediated PDT with 808 nm laser-activated PTT [87]. Copyright 2024, published by The Royal Society of Chemistry. **(C)** A diagram outlining the synthesis of CuS@COF nanosheets and the process for combined PTT/PDT therapeutic treatment [88]. Copyright 2023, published by The Royal Society of Chemistry.

Metal-organic frameworks (MOFs) and covalent organic frameworks (COFs), known for their innovative porous crystalline structures. Especially metal-organic framework materials, such as core-shell metal-organic frameworks, zinc metal-organic frameworks and their derivatives, ZIF-based materials and UIO-based metal organic frameworks, etc., have shown great potential in various cancer treatment methods such as chemotherapy, phototherapy, sonodynamic therapy, immunotherapy and gene therapy due to their unique physical and chemical properties [70–73]. This approach has improved the stability and specificity of drugs, minimized adverse effects, and contributed importantly to diagnostic medical imaging [74–78]. However, these new materials and technologies still face challenges such as cytotoxicity, human safety, and mechanism of action [79,80,81]. It is necessary to further optimize the design and synthesis methods, conduct in-depth research on their long-term effects, and formulate personalized treatment plans to address different types and stages of cancer, promote the transformation of basic research results into clinical applications, and improve the therapeutic effect of cancer [82–85]. With the advancement of science and technology and the strengthening of interdisciplinary cooperation, new treatment methods based on MOF have shown good prospects in the combined application of PDT and PTT, and are expected to bring new hope for improving the quality of life of cancer patients. In 2021, Cai and his team successfully synthesized Au@MOF core-shell hybrid materials using a layer-by-layer assembly method (Figure 6A). In this nanomaterial, the gold nanorod core generates photothermal therapeutic effects, while the porphyrin ligands in the MOF shell convert oxygen into singlet oxygen under light irradiation, enabling PDT. Additionally, the metal nodes of the MOF can catalyze the decomposition of hydrogen peroxide into oxygen, effectively overcoming the hypoxic tumor microenvironment. By modifying the material with folic acid to enhance tumor cell targeting, experiments demonstrated that Au@MOF-FA exhibits excellent biocompatibility and no significant cytotoxicity, achieving effective cancer cell killing and tumor growth inhibition through the synergistic effects of PDT and PTT [86]. Subsequently, in 2024, Jiang *et al.* constructed UCNPs@ZrMOF@ICG nanocomposites for the combined treatment of deep-seated tumors (Figure 6B). This material was synthesized by first creating a NaLuF4:Er core and its corresponding shell structure. When excited by a 1532 nm laser, upconversion nanoparticles (UCNPs) emit visible light that is absorbed by ZrMOF to trigger the production of singlet oxygen for use in PDT targeting deep tissue. Meanwhile, indocyanine green (ICG) molecules loaded into UCNPs@ZrMOF enable PTT under 808 nm laser irradiation. Experimental results showed that this combined PDT and PTT strategy effectively inhibits tumor growth [87]. To address the issue of insufficient photothermal conversion efficiency in PTT, Qian *et al.* prepared CuS@COF nanosheets in 2023 for combined PTT and PDT in tumor treatment (Figure 6C). Using CuS nanosheets as the core and employing a dual-ligand-assisted strategy, the CuS@COF nanosheets exhibited excellent near-infrared absorption properties. Experimental results demonstrated that the nanosheets generated ROS under 505 nm laser irradiation, while achieving a photothermal conversion efficiency of up to 63.4% under 1064 nm laser irradiation, with phototoxicity against 4T1 cells reaching 85.1%. *In vivo* experiments further confirmed their ability to effectively suppress tumor growth [88].

In summary, whether based on MOFs or COFs, these materials have demonstrated broad application prospects in combining PDT and PTT, providing new possibilities for developing more effective tumor treatment strategies.

### 2.7 Nanomaterials Based on Plant-Derived Photosensitizers

**Figure 7.**
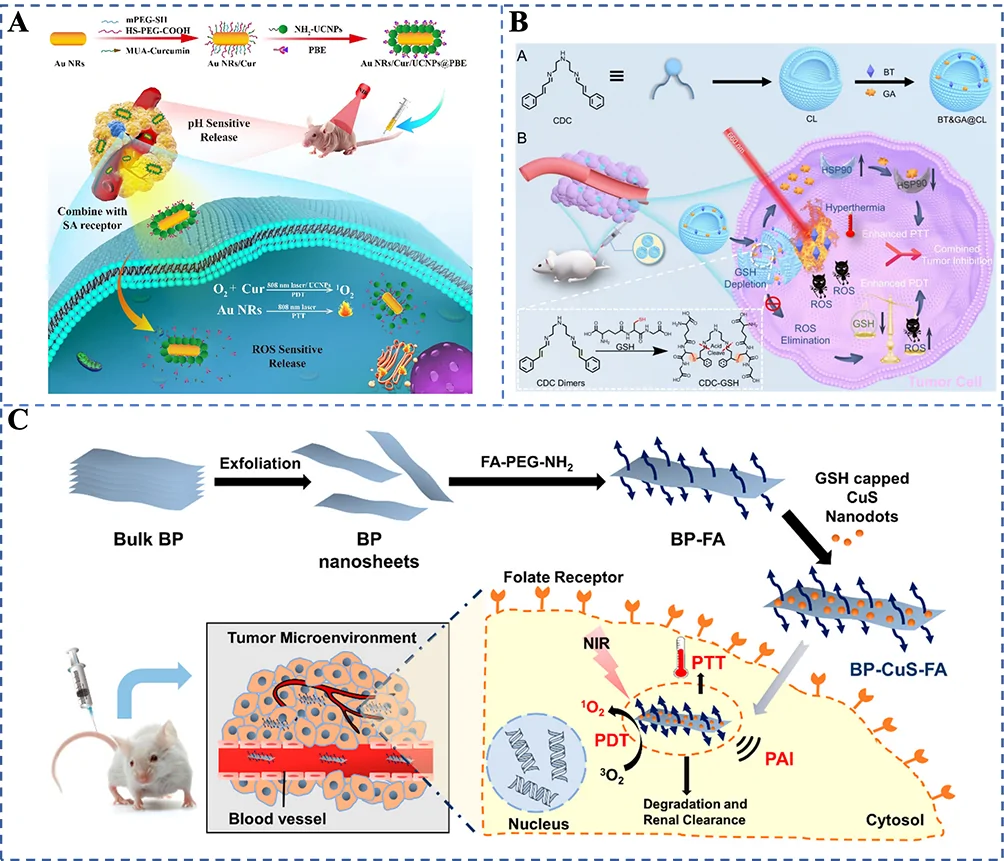
**The application of dual-mode materials Au NRs/Cur/UCNPs@PBE, BT&GA@CL and BP-CuS-FA mediating PDT and PTT in cancer treatment. (A)** Diagrams showing the assembly of Au NRs/Cur/UCNPs@PBE and their pH and ROS-responsive targeted approach against melanoma, utilizing synergistic PDT/PTT [92]. Copyright 2021, published by Elsevier B.V.. **(B)** Size distribution and TEM images of CL and BT&GA@CL [98]. Copyright 2024, published by American Chemical Society **(C)** Diagram showing the synthesis of BP-CuS-FA nanoconjugates for use in PAI-guided, tumor-targeted synergistic PDT-PTT in cancer therapy [102]. Copyright 2020, published by American Chemical Society.

Plant-based materials, such as Quince seed slime (QSM), have potential applications in many fields [89,90]. Plant-based photosensitizers, including curcumin, hypericin and phycocyanin, have attracted increasing attention in PDT and PTT due to their natural photosensitive properties. For instance, Ali and his team designed a bifunctional nanocomposite (NC) by immobilizing curcumin onto Fe3O4 nanoparticles with a silica coating, achieving a combination of PDT and PTT [91]. In 2021, Zhong *et al.* constructed a hierarchical dual-responsive cleavable nanosystem, Au NRs/Cur/UCNPs@PBE (Figure 7A), for synergistic PDT and PTT of melanoma. This system utilized gold nanorods as photothermal agents, curcumin as a photosensitizer, and upconversion nanoparticles (UCNPs) as light converters. It underwent structural evolution in the tumor microenvironment, enhancing tumor specificity and intracellular accumulation, effectively suppressing tumor growth [92]. In 2008, Chen *et al.* used hypericin as a photosensitizer in combination with epidermal growth factor receptor (EGFR)-targeted gold nanoshells as photothermal agents to explore the effects of combined PDT and PTT. The results showed that combination therapy was more effective in reducing cancer cell viability compared to either therapy alone. Additionally, phycocyanin has also proven to be a promising plant-derived photosensitizer [93]. In 2012, Liao *et al.* prepared a novel biomaterial, MWNT–CS–PC, by combining phycocyanin (PC) with carbon nanotubes for photodynamic and photothermal cancer therapy. Studies demonstrated that this composite exhibited good water solubility and stability and could effectively inhibit cancer cell growth, though further optimization is required to minimize its impact on normal cells [94]. In 2021, Zhang prepared phycocyanin (PC)-functionalized black phosphorus quantum dots (BPQDs), named PC@BPQDs, via a one-step stirring method, enhancing PTT efficiency. Research indicated that PC@BPQDs possessed excellent stability and photothermal/photodynamic performance, effectively suppressing tumor growth and showing significant potential for clinical applications [95]. Finally, in 2023, Liang’s team synthesized Mn-doped phycocyanin/polydopamine hierarchical nanostructures (MnPc@P) via a one-pot method. Under laser irradiation, this structure induced tumor cell death through PDA-mediated hyperthermia and Pc-induced reactive oxygen species generation, demonstrating superior combined PTT and PDT therapeutic effects over single-mode therapies [96]. Collectively, these studies reveal the immense potential of natural photosensitizers in modern medicine, particularly in phototherapy for cancer.

### 2.8 Nanomaterials Based on Aggregation-Induced Emission (AIE)

Aggregation-induced emission (AIE) materials are a special class of fluorescent materials that exhibit significantly enhanced luminescence in the aggregated state or solid state. Since their first introduction by Tang's team in 2001, AIE materials have demonstrated immense application potential in various fields such as bioimaging, sensors, and optoelectronic devices [97]. In recent years, the application of AIE materials in PDT and PTT has gradually increased. Deng *et al.* developed a multifunctional nanocomposite, BT&GA@CL (Figure 7B), for combined PDT and PTT in cancer treatment. This composite consists of cinnamaldehyde dimer self-assembled into Cinnamaldehyde liposome (CL), which can deplete glutathione (GSH) and responsively release drugs. The released BT aggregates function simultaneously as photothermal agents and photosensitizers, while gambogic acid (GA) inhibits the expression of heat shock protein HSP90. Experiments showed that BT&GA@CL not only effectively downregulated intracellular GSH levels and generated reactive oxygen species (ROS), but also suppressed HSP90 expression, thereby enhancing the efficacy of PDT and PTT [98].

Tetraphenylethylene (TPE), a typical AIE material, possesses many advantages; however, its poor photothermal therapeutic performance has limited its practicality. Boron-dipyrromethene (BODIPY), an aggregation-caused quenching (ACQ) material, can be used in PDT and PTT, but its fluorescence diminishes or even quenches at high concentrations or in the aggregated state, affecting singlet oxygen generation. In 2021, Shi Ruijie's team constructed an intermolecular system, D - π…π' - A, composed of tetraphenylethylene (TPE) and boron-dipyrromethene (BODIPY), achieving synergistic effects in PDT and PTT [99]. The study found that specific combinations, such as TPA-BOD, performed exceptionally well in PDT synergy, providing new insights into the integration of AIE and ACQ materials. In 2024, Guo *et al.* synthesized α-α linked bis-BODIPY derivatives containing TPE (TPEB and 2TPEB). These materials exhibit near-infrared absorption and AIE activity. When self-assembled into nanoparticles with the surfactant F-127, they localize in lysosomes and efficiently generate ROS. Experiments demonstrated that 2TPEB NPs not only achieved high photothermal conversion efficiency (η ~68.3%) but also exhibited significant antitumor activity. Compared to the commercial reagent indocyanine green (ICG), 2TPEB NPs showed lower dark toxicity and higher phototoxicity, demonstrating strong potential for clinical applications [100].

### 2.9 Other Materials

In recent years, a variety of inorganic materials have demonstrated great potential in PDT and PTT. For instance, Li *et al.* proposed the F127@CNs-CuS/MnO2 multifunctional nanoplatform, while Jana’s team designed the BP-CuS-FA (Fig. 7C) biocompatible nanoconjugate [101,102]. Dong *et al.* reviewed the applications of black phosphorus, and Song explored the use of molybdenum disulfide [103,104]. Additionally, Han developed the PHPD-NPs composite material, and Subramaniyan Bharathiraj’s team synthesized PPy@BSA-Astx nanoparticles [105,106]. N. Sanoj Rejinold highlighted the potential of semiconductor polymer nanoparticles, and Gui *et al.* developed magnetically targeted capacitive heterostructures based on polypyrrole [107,108]. In summary, many types of materials have been developed to be the dual-mode platform mediating PDT and PTT. However, each type of material has its strength and weakness. In order to have a clearer understanding of these materials, the advantages and disadvantages of above dual-mode materials are analyzed and summarized in Table 2.

## 3. Dual-Mode Photosensitive materials Mediating PDT and CDT

CDT is a treatment method based on Fenton or Fenton-like reactions, which utilizes transition metal ions (such as Fe2+/Fe3+) to catalyze the decomposition of hydrogen peroxide (H2O2) into highly toxic hydroxyl radicals (·OH), thereby damaging tumor cells. The Fe2+/Fe3+ system, in the presence of photons, is a Fenton process whose activity is highly dependent on pH [109–112]. A significant advantage of CDT is its ability to work effectively in hypoxic environments without requiring external light activation, making it particularly attractive for treating deep-seated tumors and tumors in hypoxic conditions [113]. Combining PDT with CDT not only attacks tumor cells through two distinct mechanisms, enhancing therapeutic efficacy, but also compensates for the reduced efficiency of PDT in hypoxic environments. Thus, the combination of PDT and CDT provides a new strategy to overcome the limitations of PDT under hypoxic tumor conditions and achieve more effective cancer treatment.

**Table 2.** The advantages and disadvantages of different dual-mode materials mediating PDT and PTT.

Materials	Advantages	Limitations	Ref.
**Materials Based on Porphyrin Photosensitizers**	- High biocompatibility and low toxicity ensure safety. - SERS and NIR enable real-time monitoring and immune enhancement for improved therapy	- The depth of tissue penetration is limited. - Large-scale production is complex. - Clinical application needs to be optimized.	[18–26]
**Materials Based on Chlorin (Ce6) Photosensitizers**	- Ph-sensitive Ce6 release achieves precise targeting and is guided by dual-modal imaging for treatment. - Deep tissue penetration under the NIL-II window.	- Large-scale production is complex. - Information on long-term toxicity and biological distribution is limited.	[27–41]
**Materials Based on Metal Complex Photosensitizers**	- Achieved complete tumor ablation without recurrence. - Bimodal imaging (MRI and PAT) provides precise treatment guidance.	- The potential clinical translation challenges, long-term toxicity and biodistribution information are limited. - Large-scale production is complex.	[42–49]
**Materials Based on Cyanine Dye Photosensitizers**	- Real-time imaging guides the precise focusing of the laser beam on the tumor area,	- ICG has poor stability, is prone to photobleaching, and has a medium photothermal conversion efficiency.	[50–57]
**Materials Based on Other Organic Dye Photosensitizers**	- Early PTT/PA characteristics, late PDT/fluorescence imaging, MTX targeted delivery, wide absorption range.	- Large-scale production is complex. - The long-term toxicity and biological distribution information is limited。	[58–69]
**Materials Based on Organic Framework Photosensitizers**	- Deep tissue penetration is achieved through NIL-IIB light. -High ROS generation rate, significant tumor suppression.	- Large-scale production is complex. - Information on long-term toxicity and biological distribution is limited.	[86–88]
**Materials Based on Plant-Derived Photosensitizers**	- Effectively inhibit the growth of tumors in the body with the least systemic toxicity.	- Large-scale production is complex. - Information on long-term toxicity and biological distribution is limited.	[91–96]
**Materials Based on Aggregation-Induced Emission**	- Expand to the NIR range to enhance deep tissue penetration. - AIE activity enhances fluorescence imaging and effectively targets lysosomes for cancer treatment.	- It needs to be optimized to achieve *in vivo* application throughout the body. - Large-scale production is complex. - Information on long-term toxicity and biological distribution is limited.	[98–100]
**Other Materials**	- Folate receptor targeted delivery, renal clearance after photodegradation to reduce toxicity. - Produce a "dual killing" effect, inducing an anti-cancer immune response.	- Large-scale production is complex. - Information on long-term toxicity and biological distribution is limited.	[101–108]

**Figure 8.**
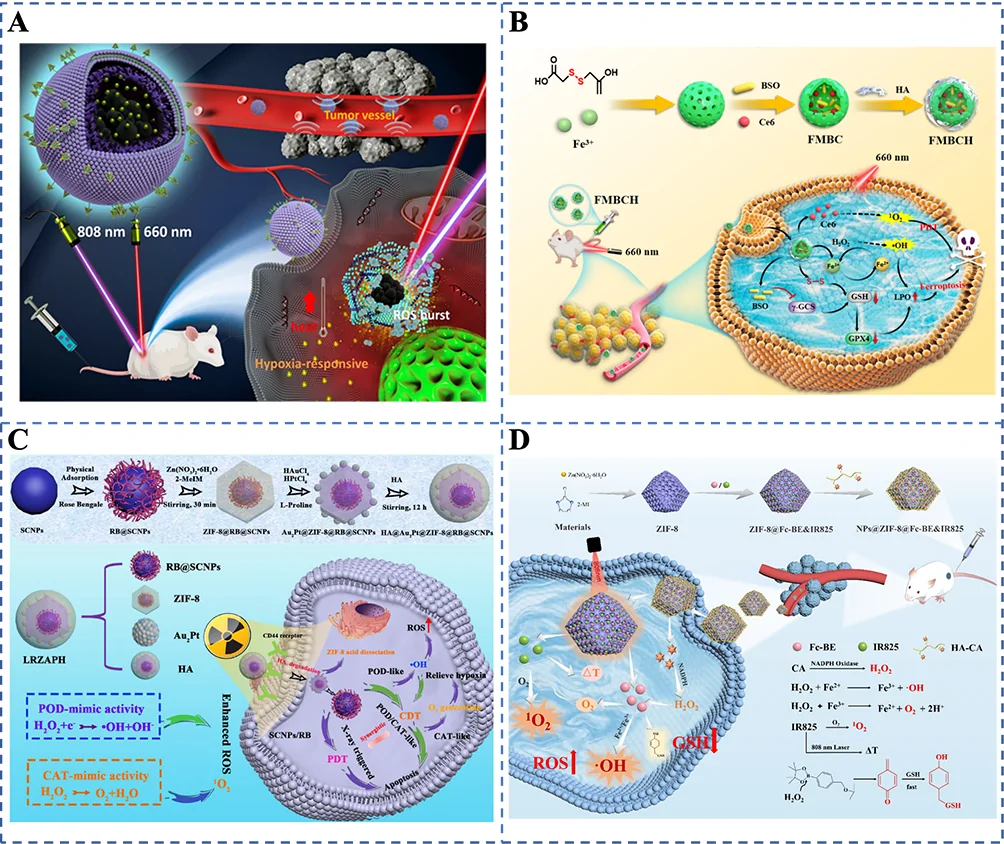
**The application of dual-mode materials mediating PDT and CDT in cancer treatment. (A)** Diagram showing a hypoxia-activated ROS burst therapy triggered by coirradiation with 808/660 nm light [114]. Copyright 2020, published by Elsevier B.V.. **(B)** Design schematic of FMBCH and its synergistic CDT/PDT [119]. Copyright 2024, published by American Chemical Society. **(C)** Diagram illustrating the versatile LRZAPH nanocatalytic platform and its synergistic X-PDT/CDT mechanism for treating tumors [123]. Copyright 2024, published by Elsevier B.V.. **(D)** Development of nanoparticles for triply enhanced CDT/PDT through photothermal treatment, H_2_O_2_ elevation, and GSH consumption by accelerating and boosting oxidative stress amplification [127]. Copyright 2023, published by American Chemical Society.

In materials of mediating the combination between PDT and CDT, Ce6 has been widely used as a photosensitizer. In 2020, Tang *et al.* synthesized amphiphilic block copolymers mPEG-P(Glu-MN) via a three-step process to prepare hypoxia-activated ROS burst liposomes, SPIOCs/Ce6@P(MNs)RGD (Figure 8A). These liposomes dissociate in the hypoxic tumor microenvironment, generating singlet oxygen via PDT and hydroxyl radicals via CDT, significantly suppressing malignant glioma growth [114]. In 2021, Chen *et al.* developed multifunctional nanoparticles (HCNPs) self-assembled from Ce6 and heme, which generate hydroxyl radicals (•OH) and deplete intracellular glutathione (GSH) in the tumor environment while simultaneously enhancing both PDT and CDT effects and inducing ferroptosis [115]. Additionally, in 2022, Mo *et al.* designed and synthesized the multifunctional nano-platform MnO_2_@NH_2_-MIL101(Fe)@Ce6-F127 (MNMCF), which capable of effectively ablating tumors without issues of poor tolerance or long-term side effects, demonstrating potential for cancer therapy [116]. Liu *et al.* constructed a tumor pH-responsive self-catalytic nanoreactor (CESAR) based on copper peroxide using an albumin-mediated biomimetic mineralization strategy, further enhancing Ce6-based PDT effects [117]. In 2022, Hu *et al.* prepared a multifunctional nanoplatform, Ce6/FeOOH@BSA, for efficient delivery of the photosensitizer Ce6, broadening the biomedical applications of nanomaterials [118]. In 2024, An’s team and Yan’s team advanced this field by developing biodegradable nanocomposites, Fe(SS)-MOF@BSO/Ce6@HA (FMBCH) (Figure 8B) and HAMnO_2_A, enabling synergistic PDT/CDT with excellent tumor-targeting capabilities and anti-tumor efficacy [119,120].

Various organic dye-based photosensitizers have shown significant effects in PDT and CDT. Methylene blue (MB) and rose bengal (RB) are organic dyes that generate ROS upon exposure to specific wavelengths of light, causing cellular damage. This property makes them valuable tools in PDT. In 2021, Hu *et al.* developed a supramolecular photosensitizing system, OCZWM, using methylene blue (MB) as the photosensitizer, exhibiting excellent O2 loading and pH-responsive release properties. It demonstrated significant toxicity against hepatocellular carcinoma cells under hypoxic conditions, greatly improving PDT efficiency [121]. In the same year, Zou *et al.* designed a core-shell nanostructure, NaYF₄:20%Yb, 2%Er@Cu/ZIF-8/RB@F127 (UCZRF), which effectively enhanced ROS levels and anti-tumor effects with good biocompatibility [122]. Similarly, Liu *et al.* prepared a multifunctional X-ray-triggered nanoplatform, LRZAPH in 2024 (Figure 8C), showing excellent tumor-killing and long-term suppression effects [123]. The teams of Huang and Qin developed multifunctional nanoparticles (NPs) based on zinc phthalocyanine (ZnPc): FeP-ZnPc and ZnPc@PDA-Fe2+ for synergistic near-infrared-triggered PDT/CDT while depleting glutathione (GSH) [124,125]. *In vitro* experiments demonstrated that the combined therapy outperformed single-mode PDT in HeLa cells, showcasing its potential in cancer treatment.

Li and Yang *et al.* respectively prepared IR780- and IR825-based photosensitizers for synergistic PDT/CDT: ZHTC@IR780 NPs and NPs@Fc-BE&IR825 (Figure 8D) [126,127]. ZHTC@IR780 NPs possess catalase-like and glutathione peroxidase-like activities, catalyzing the decomposition of endogenous H2O2 to produce O2, alleviating tumor hypoxia, and generating •OH via Fenton-like reactions while depleting GSH, effectively promoting tumor cell apoptosis, providing an innovative strategy for synergistic cancer therapy. NPs@Fc-BE&IR825 is an integrated nanosystem that demonstrated excellent anti-tumor effects in both *in vitro* and *in vivo* experiments with high biosafety. Recently, Yuan *et al.* designed and synthesized a small-molecule BODIPY-ferrocene conjugate, BDP2IFc, which kills tumor cells via CDT in the dark and significantly enhances anti-tumor efficiency by combining PDT and CDT under illumination, offering a new approach for synergistic cancer therapy [128]. Jasim, Cheng, and Chen also developed CPNPs (EDN3-CP nanocomposites), conjugated polymer nanoparticles (PPE-Cu NPs), and a tumor-targeting polymer nanohybrid, TSM, respectively, all of which effectively combined PDT and CDT for cancer treatment [129–131].

In 2022, Li *et al*. developed Mn (II) ion and pyrophosphorus-A (PPa) -modified iron oxide nanoparticles, named IMOP [132]. In the same year, Cheng *et al*. designed MnO2 nanoplate-mediated universal probes (MNSGP). The research on IMOP emphasized its unique oxygen production capacity and GSH consumption characteristics. This is helpful to alleviate the hypoxic environment of tumors and enhance the therapeutic effect of PDT. The application of IMOP in mouse models demonstrated a significant inhibitory effect on tumor growth, with a relatively small impact on normal tissues. MNSGP not only realizes efficient CDT/PDT synergistic therapy, but also has the dual miRNA detection function, enhancing its application potential in the field of precision medicine. MNSGP has demonstrated excellent cytotoxicity and selectivity in both *in vitro* and *in vivo* experiments [133]. In 2023, Yang *et al.* proposed an H2O2 upregulation strategy to enhance PDT and CDT, synthesizing pH-responsive Fenton reagents (Fc-CA) encapsulated in hyaluronic acid-coated porphyrin-based metal-organic frameworks to form nanoparticles (Fc-CA-PCN-HA). These nanoparticles activate NADPH oxidase to increase H2O2 levels, catalyze the generation of •OH and O2, alleviate tumor hypoxia, enhance PDT/CDT efficacy, and exhibit excellent safety and biocompatibility [134]. In 2024, Raj *et al.* prepared two-dimensional nanosheets loaded with peroxidase-mimicking DNAzymes, demonstrating promising therapeutic activity [135].

## 4. Dual-Mode Photosensitive materials Mediating PDT and GT

Specific gases, including oxygen, nitric oxide, carbon monoxide, and others with therapeutic effects, have been utilized in GT for the treatment of tumors. The key feature of GT is its ability to specifically regulate the tumor microenvironment, particularly by improving local oxygen content, thereby enhancing the efficacy of PDT. Adequate oxygen is not only essential for generating ROS during PDT but also improves the activity of immune cells. Additionally, certain gases possess independent anti-tumor effects, directly inhibiting tumor growth or inducing apoptosis in cancer cells. Therefore, combining PDT with GT not only addresses the issue of reduced PDT efficacy in hypoxic environments but also enhances treatment outcomes by optimizing oxygen supply in tumor regions [136].

**Figure 9.**
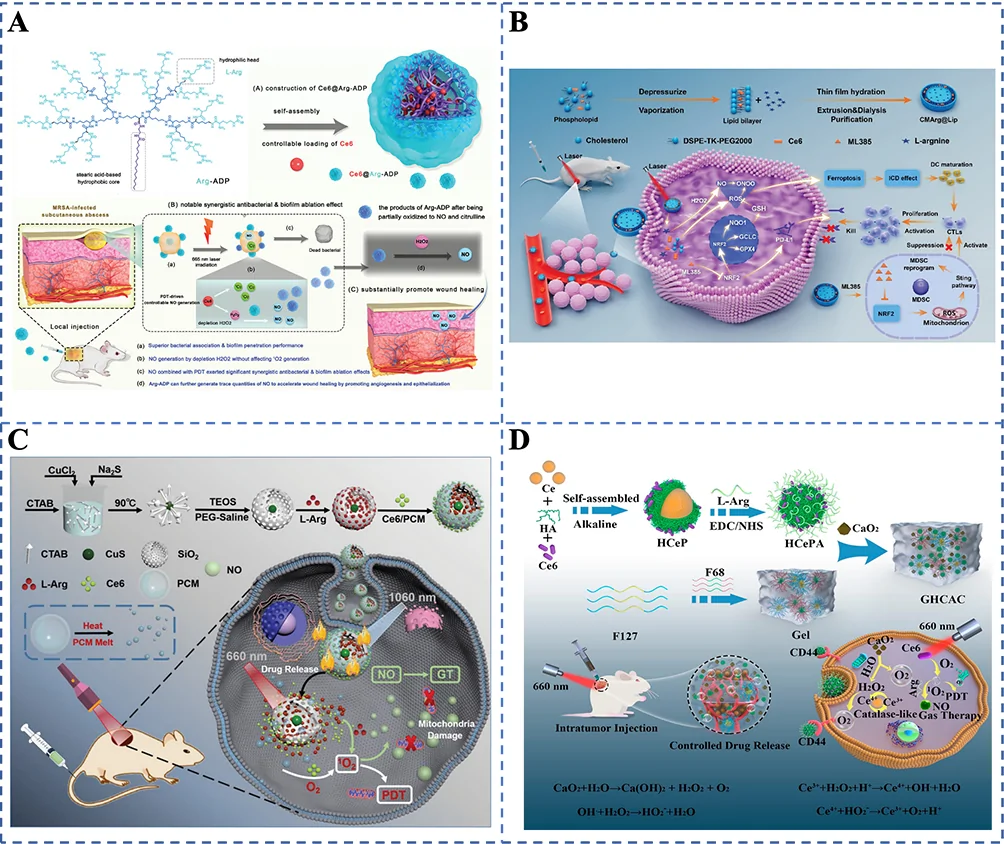
**The application of dual-mode materials mediating PDT and GT in cancer treatment. (A)** Diagram showing the preparation of Ce6@Arg-ADP and the associated mechanisms for highly efficient synergistic antibacterial PDT/NO therapy and promotion of wound healing [137]. Copyright 2021, published by Wiley-VCH GmbH. **(B)** Diagram showing the PDT-GT facilitated by CMArg@Lip for reversing the tumor's immunosuppressive microenvironment [139]. Copyright 2024, published by Wiley-VCH GmbH. **(C)** Diagram illustrating the structure of CuS@SiO2-l-Arg@PCM-Ce6 nanoparticles for thermally controlled drug release and synergistic PDT/GT [140]. Copyright 2021, published by Wiley-VCH GmbH. **(D)** Diagram showing oxygen-generating hydrogels (GHCAC) designed to overcome tumor hypoxia, thereby enhancing PDT/GT [142]. Copyright 2022, published by American Chemical Society.

In 2021, Zhu *et al.* reported an antibacterial strategy combining PDT and nitric oxide (NO). They developed a Ce6@Arg-ADP system (Fig. 9A) using L-Arg-rich amphiphilic dendritic peptides (Arg-ADP) as carriers. This system exhibited excellent bacterial binding and biofilm penetration capabilities. It utilized H2O2 generated during PDT to produce large amounts of NO without compromising singlet oxygen generation, significantly enhancing antibacterial and biofilm removal effects while promoting wound healing [137]. Fu *et al.* developed the Cy-NMNO@SiO2 nanoplatform for antibacterial applications and skin cancer treatment [138]. Wang’s team designed the CMArg@Lip multifunctional nanoplatform (Fig. 9B) for cholangiocarcinoma treatment. This system incorporates the photosensitizer Ce6, the NO-producing agent L-arginine, and the NRF2 inhibitor ML385. It addresses the difficulties presented by the hypoxic conditions and antioxidant microenvironments of tumors, induces ferroptosis in cancer cells, and boosts immune responses [139]. Zhu *et al.* proposed a strategy based on thermally controlled multishell nanoparticles (CuS@SiO_2_-l-Arg@PCM-Ce6) (Figure 9C). Upon laser irradiation, the phase-change material (PCM) melted, releasing Ce6 and L-Arg. Ce6 generated singlet oxygen for PDT, while oxidizing L-Arg to release NO, achieving precise drug release and effective anti-tumor efficacy [140].

Wan *et al.* developed a biomimetic multifunctional nanosystem, L-Arg@PCN@Mem, in 2018. This system utilized cancer cell membranes to encapsulate PCN loaded with the NO donor L-Arg. When exposed to near-infrared (NIR) light, it simultaneously enabled PDT and oxidized L-Arg to generate NO [141]. Zhang’s team developed a sustained oxygen-supplying system (GHCAC) (Fig. 9D) based on gels and cerium nanoparticles. This system continuously produced oxygen through calcium peroxide embedded in a thermosensitive hydrogel and a nanocarrier, HCePA, alleviating tumor hypoxia and oxidizing L-arginine into nitric oxide for synergistic GT, achieving a "single injection, multiple irradiations" treatment strategy [142]. In 2024, Peng *et al.* designed a light-activated, controllable NO-producing multifunctional nanosystem called Ru-PArg-HA, aimed at synergistic photodynamic and NO cancer therapy. Hyaluronic acid (HA) served as both the scaffold and targeting moiety, poly-L-arginine acted as the NO donor, and [Ru(phen)2(PIP-OCH3)]2+ was used as the photosensitizer. Under 470 nm light irradiation, this system catalyzed NADH oxidation to generate ROS for PDT and converted arginine in situ to produce NO for GT [143].

Feng *et al.* proposed a glutathione (GSH)-responsive biodegradable "nanobomb" strategy for cancer treatment. They developed the "nanobomb" L-Arg/MB@MP by embedding methylene blue (MB) and L-arginine (L-Arg) within polyethylene glycol-modified mesoporous organosilica nanoparticles (MONs). This nanoplatform showed an extended circulation time and superior tumor accumulation. It mitigated tumor hypoxia, induced immunogenic cell death, and reversed the immunosuppressive microenvironment, offering novel perspectives on combining PDT with GT for treating hypoxic solid tumors [144]. Similarly, Xia *et al.* in 2022 developed a GSH-responsive NO-generating nanosystem, Nic-MOF@HA, to enhance PDT efficacy. By encapsulating nicorandil into porous porphyrin-based metal-organic framework nanoparticles and electrostatically absorbing hyaluronic acid onto their surface, the system released nicorandil upon degradation in the tumor site, reacting with GSH to produce NO, depleting GSH, and increasing oxygen supply to promote ROS generation [145]. Du *et al.* in 2022 proposed a system, PBF-g-CO/PEG, for NIR-II fluorescence imaging-guided photodynamic/CO gas synergistic cancer therapy. By conjugating the CO donor CORM-401 to water-soluble polyamidoamine dendrimer-modified NIR-II fluorescent conjugated polymer brushes, they achieved excellent anti-cancer effects [146].

**Figure 10.**
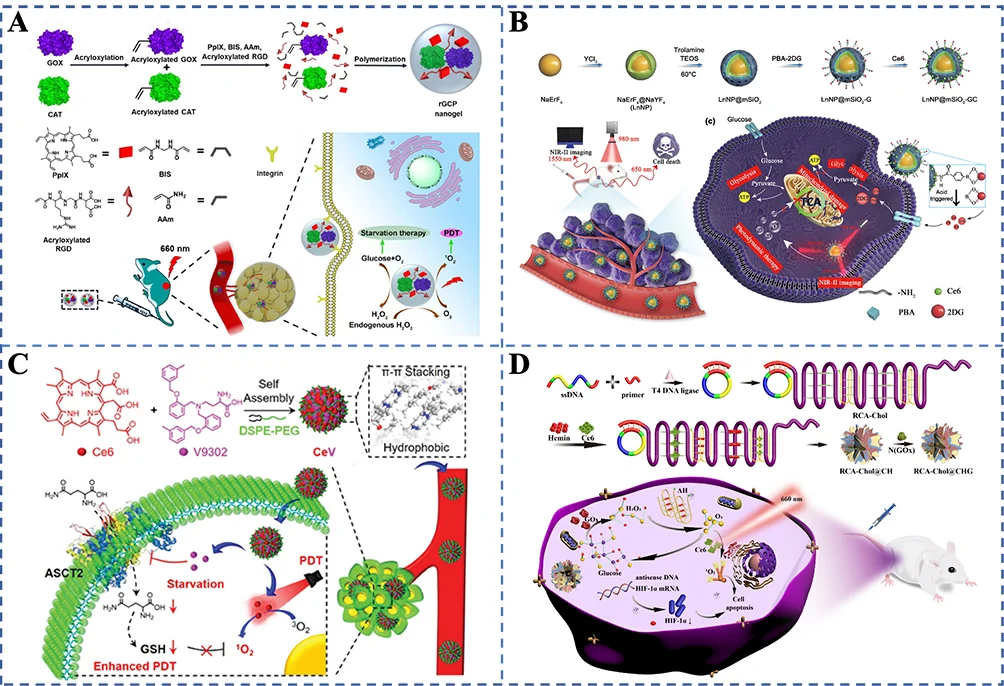
**The application of dual-mode materials mediating PDT and ST in cancer treatment. (A)** The preparation method of rGCP nanogels and their use in enhanced synergistic cancer therapy by integrating ST and PDT [150]. Copyright 2022, published by Elsevier Ltd. **(B)** Diagram illustrating precise tumor-starvation therapy guided by NIR-II fluorescence imaging, achieved through interference with glucose metabolism reprogramming [153]. Copyright 2024, published by Elsevier B.V. and Science China Press. **(C)** Chemical structures of Ce6 and V9302, and the method of preparing CeV *via* self-assembly through intermolecular *π–π* stacking and hydrophobic interactions. Also illustrated is the proposed mechanism by which CeV enhances PDT through glutamine starvation [154]. Copyright 2021, published by Wiley-VCH GmbH. **(D)** Synthesis pathway of the 'Domino' cascade reactor RCA-Chol@CHG based on a DNA hydrogel, and a diagram illustrating the regulatory mechanism of combined ST and PDT [155]. Copyright 2023, published by Elsevier Masson SAS.

## 5. Dual-Mode Photosensitive materials Mediating PDT and ST

ST kills tumor by inhibiting tumor angiogenesis or blocking nutrient supply pathways, thereby inducing "starvation" in tumor cells to suppress their growth and metastasis. When combined with PDT, ST can enhance the sensitivity of tumor cells to PDT by altering the tumor cells status. More importantly, it alleviates the oxygenation effects caused by high-density vasculature within tumors, making hypoxic regions—previously resistant to PDT—more responsive to treatment. Additionally, by inhibiting tumor cell proliferation, ST reduces the ability of tumor cells to repair damage caused by PDT, further enhancing its efficacy. Thus, the combination of PDT and ST not only complements the strengths of each approach but also addresses the limitations of PDT in hypoxic environments [147].

In 2021, Liu *et al.* prepared a biomimetic cascade polymer nanoreactor, GOx/CAT-NC, which utilized glucose oxidase (GOx) and catalase (CAT) to catalyze the production of oxygen from endogenous H2O2 while accelerating glucose decomposition, promoting the generation of cytotoxic singlet oxygen [148]. In the same year, Luo constructed a mitochondria-targeted composite enzyme nanogel featuring O2 and H2O2 recycling. This system precisely targeted the mitochondria of breast cancer cells and enhanced PDT efficacy by improving the tumor hypoxic environment through cascade catalysis [149]. Fan developed rGCP nanogels (Fig. 10A), which exhibited self-oxygen-supplying capabilities, significantly enhancing the synergistic anticancer effects of ST and PDT [150]. Wan designed a porphyrin covalent organic framework enzyme nanopocket, achieving long-term synergistic effects between ST and PDT, effectively inhibiting tumor growth with excellent biosafety [151]. Liu developed the multifunctional composite CPCG, which improved tumor hypoxia through the dual enzymatic activity of CeO2 nanoparticles while consuming glucose for ST [152]. Wu proposed the LnNP@mSiO2-GC nanoplatform (Fig. 10B), which not only monitored the accumulation of nanomaterials in tumors but also enhanced the efficiency of 2DG-mediated ST [153]. Zhao developed the CeV (Fig. 10C) nanodrug, which downregulated ASCT2 expression to block glutamine metabolism, reduced intracellular GSH levels, and enhanced PDT [154]. Liu constructed the DNA hydrogel "domino" cascade reactor RCA-Chol@CHG (Fig. 10D), achieving synergistic enhancement of PDT and ST through a series of catalytic reactions [155]. Lv designed the YMIGL theranostic agent, which not only penetrated the blood-brain barrier to target orthotopic glioma cells but also efficiently inhibited tumors through the synergistic effects of ST and PDT [156]. These studies demonstrated that the combination of PDT and ST is an effective strategy for tumor treatment.

## 6. Dual-Mode Photosensitive Materials for PDT and PAT

PAT is a treatment method that utilizes specific photosensitive compounds to generate acidic substances under light irradiation, causing local pH decrease and thereby disrupting the intracellular homeostasis of cancer cells [157]. Its characteristic lies in the ability to specifically alter the acid-base balance of the tumor microenvironment, inhibiting tumor cell metabolism and proliferation, and even directly inducing apoptosis. By reducing the pH in tumor regions, PAT enhances the permeability of tumor cell membranes, which not only helps improve tumor cell uptake of photosensitizers but also increases cellular sensitivity to ROS, thereby enhancing the efficacy of PDT. Additionally, PAT can alleviate the hypoxic state within tumors, which is an important limiting factor for PDT efficacy since sufficient oxygen is essential for ROS generation during PDT. Therefore, by adjusting the acid-base balance of the tumor microenvironment, PAT provides a new strategy to address the inefficiency of PDT in hypoxic environments. This combined therapy opens new possibilities for cancer treatment, particularly showing potential value in combating tumors resistant to conventional therapies.

**Figure 11.**
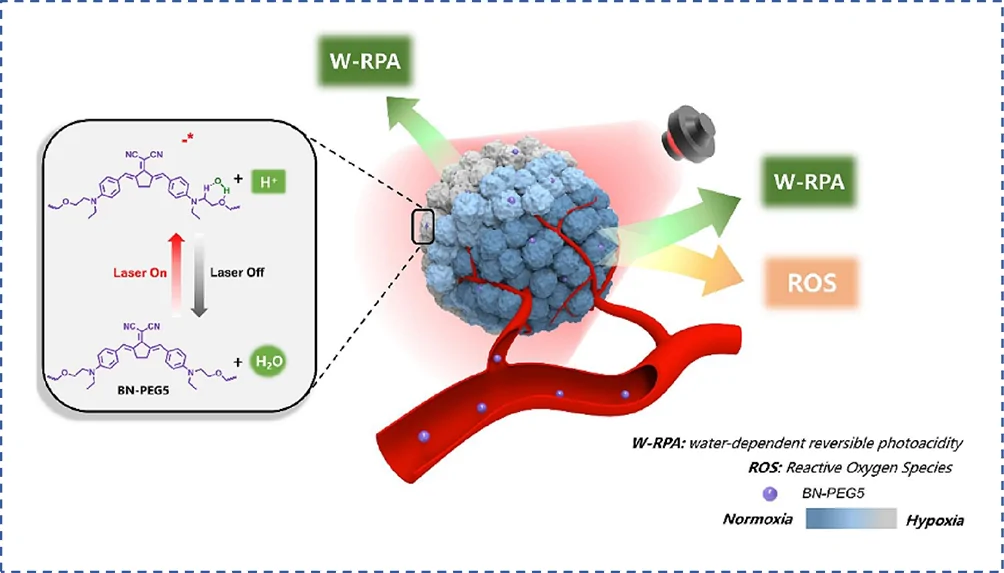
**Diagram showing the combined PDT and water-dependent reversible PAT facilitated by BN-PEG5** [158]. Copyright 2023, published by Elsevier B.V.

In 2023, Kang *et al.* constructed the cyclopentanone photosensitizers (B-PEG5) with two characteristics - photoinduced intramolecular charge transfer (ICT) and hydrophilic segment electron donors - exhibited water-dependent reversible photoacidity (W-RPA) properties. Based on this structure, they designed and synthesized cyclopentylmalononitrile dye (BN-PEG5) (Figure 11A), which produce ROS and H+ under 635 nm laser irradiation, BN-PEG5 could showing strong phototoxicity against various tumor cells under both normoxic and hypoxic conditions, with its IC50 value under hypoxia comparable to that of commercial drug HpD under normoxia, demonstrating the advantages of combined PDT and W-RPAT for treating hypoxic tumors [158]. In 2024, their team designed and synthesized a porphyrin derivative, TPP-O-PEG5, which combines the properties of PDT and water-dependent reversible photoacid therapy (W-RPAT) and developed its nanoparticle form [159]. The TPP-O-PEG5 NPs are capable of simultaneously generating ROS and H+ ions when exposed to 660 nm laser irradiation. This makes them highly phototoxic to various tumor cell lines under both normoxic and hypoxic conditions, with no significant difference in their half-maximal inhibitory concentration (IC50) values. *In vivo* experiments showed that its therapeutic effect on large hypoxic tumors was superior to clinical photosensitizer, NPe6, offering a novel approach for porphyrin photosensitizers to overcome the limitations of hypoxic environments and enhance phototherapeutic effectiveness for tumors in hypoxic conditions.

## 7. Multimodal Photosensitive Materials for the combination of PDT, PTT and CDT

PDT uses photosensitizers to generate ROS under light irradiation to destroy cancer cells; PTT relies on photothermal conversion materials to absorb light energy and convert it into heat, directly destroying tumor tissue; CDT utilizes Fenton or Fenton-like reactions to produce highly toxic hydroxyl radicals (•OH), which remain effective even in hypoxic environments. The trimodal combination therapy of PDT, PTT and CDT not only addresses the limitations of PDT in hypoxic environments and deep-seated tumors, but also utilizes the heat from PTT to increase tumor permeability and ROS diffusion range, while leveraging CDT's effectiveness under hypoxia to ensure multi-directional attacks on tumor cells. Therefore, the combination of PDT, PTT and CDT not only enhances the advantages of each therapy but also provides new strategies for improving the tumor microenvironment, increasing treatment depth, opening more effective pathways for cancer treatment [160]. The multimodal photosensitive materials for the combination of PDT, PTT and CDT are summarized in Table 3.

In 2020, Han *et al.* developed a near-infrared-responsive multifunctional nanoparticle, ICG-MSN-Fe-CD. *In vitro* experiments revealed that the combined CDT/PTT/PDT strategy significantly surpassed single CDT or dual PTT/PDT in suppressing HeLa cells, highlighting its promising potential for integrated cancer therapy [161]. In 2021, more multimodal photosensitive materials were designed and displayed synergistic PTT/PDT/CDT effects [162,163,164]. In 2023, Lv *et al.* designed a tumor microenvironment-responsive multifunctional nanoplatform Bi2S3@Bi@PDA-HA/Art NRs, providing new ideas for combining phototherapy with traditional Chinese medicine monomers to treat hypoxic tumors [165]. In the same year, Ma *et al.* proposed the multifunctional nanoplatform HMPB@TCPP-Cu for combined PTT, PDT and CDT of hepatoblastoma, which effectively inhibited tumor growth with minimal biotoxicity, offering new strategies to overcome thermal resistance and enhance heat-related therapies [166]. Wang and colleagues developed the FA-PPC nanoplatform, where Pt nanocrystals alleviated tumor hypoxia to promote PDT/PTT, while GSH-activated Cu2+ release triggered Fenton reactions to produce ROS and enhance CDT [167].

**Figure 12.**
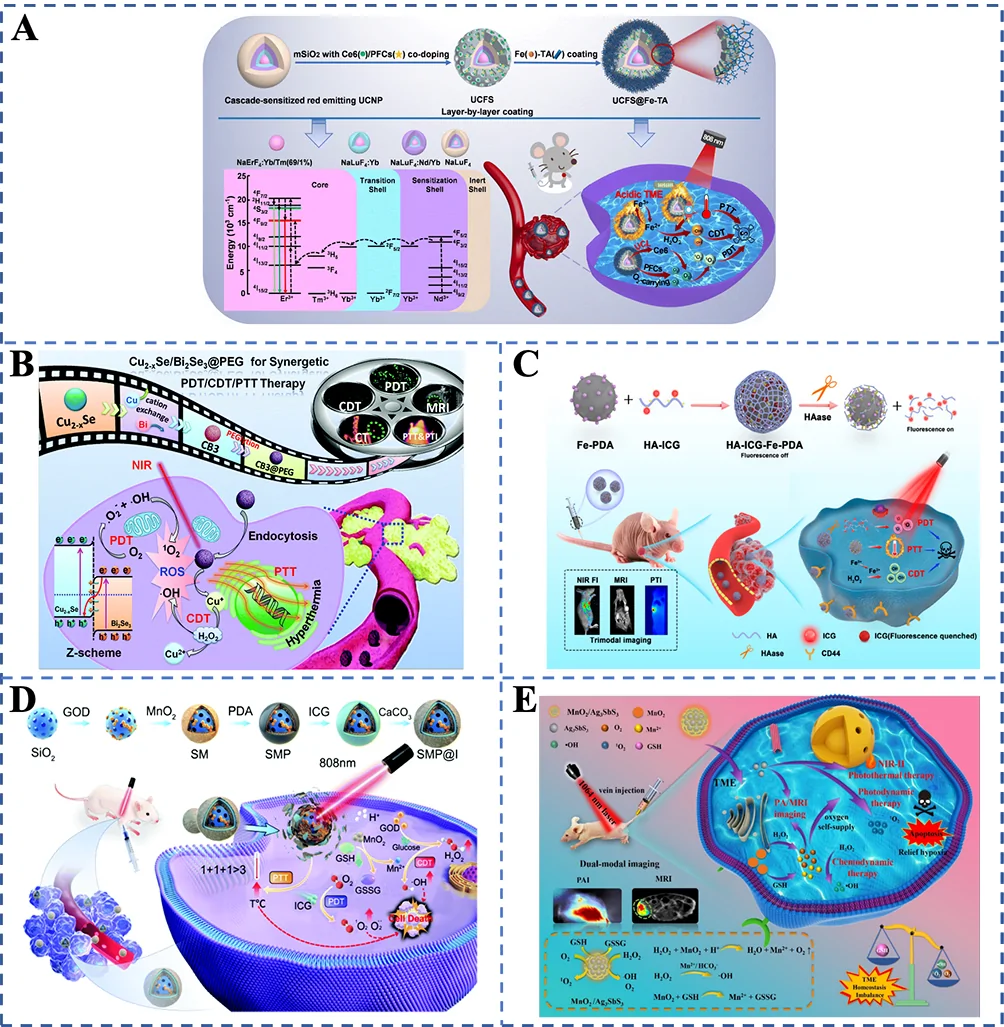
**The application of dual-mode materials mediating PDT, PIT and CDT in cancer treatment. (A)** Diagram illustrating the manufacturing process of UCFS@Fe-TA, the proposed energy transfer mechanisms in Nd3+-sensitized red-emitting UCNPs, and the operational mechanism of the UCFS@Fe-TA nanoplatform for simultaneous multimodal tumor therapies triggered by single 808 nm irradiation [168]. Copyright 2023, published by The Royal Society of Chemistry. **(B)** Diagram showing the preparation method and multimodal imaging-guided cancer theranostics of Cu2−xSe/Bi2Se3@PEG [171]. Copyright 2021, published by The Royal Society of Chemistry. **(C)** Development of HA-ICG-Fe-PDA and its application in multimodal imaging-guided CDT, PDT, and PTT for treating CD44-overexpressing triple-negative breast cancer (TNBC) [172]. Copyright 2023, published by American Chemical Society. **(D)** Diagram showing the synthesis process and the antitumor mechanism of SMP@I [174]. Copyright 2022, published by The Royal Society of Chemistry. **(E)** Diagram illustrating the synthesis process and therapeutic mechanism of PAI/MRI-guided oxygen self-supply PDT/CDT/PTT combined therapy within the NIR-II biowindow [177]. Copyright 2022, published by American Chemical Society.

Additionally, some studies have enhanced PDT efficacy by incorporating upconversion luminescence or cascade sensitization mechanisms. Li's team designed the UCFS@Fe-TA nanoplatform (Figure 12A), utilizing Nd3+→Yb3+→Tm3+→Er3+ cascade-sensitized upconversion luminescence to enhance PDT effects, with the Fe-TA network exhibiting high photothermal conversion efficiency and Fenton catalytic activity [168]. In 2024, Zhang *et al.* prepared dual-source-driven parachute-like Au2Pt@PMO@ICG Janus nanomotors (APIJNS) for cancer treatment. The Au2Pt bimetallic nanozyme exhibited CAT-/POD-like catalytic activity, decomposing excess H2O2 in the tumor microenvironment to produce O2 and ·OH, where O2 could be converted by ICG into 1O2 to enhance PDT/CDT. Meanwhile, PTT triggered by APIJNS significantly boosted POD-like activity, enabling efficient synergistic cell killing and self-thermophoretic propulsion. The chemical/NIR-driven mode enabled APIJNS to rapidly accumulate and penetrate tumor lesions, enhancing the trimodal synergistic efficacy of PDT/PTT/CDT [169].

To further improve the precision of treatment, many studies have also integrated multimodal imaging capabilities in multifunctional nanoplatforms. For example, Wang prepared Au2Pt-PEG-Ce6 nanozymes in 2020, which enhanced PDT and CDT effects through their catalase and peroxidase-like activities respectively, while exhibiting strong near-infrared absorption for PTT and possessing triple-modal imaging capabilities (photoacoustic, photothermal and X-ray computed tomography) [170]. In 2021, Wang *et al.* prepared Cu2-xSe/Bi2Se3@PEG (CB3@PEG) nanoheterostructures (Figure 12B). CB3@PEG exhibited enhanced near-infrared absorption and photothermal conversion efficiency (60.4%), capable of producing hydroxyl radicals and singlet oxygen for PDT. Cu ions-initiated Fenton reactions, endowing it with CDT capability and T1-weighted magnetic resonance imaging (MRI) capacity. Furthermore, the Bi element provided computed tomography (CT) imaging capability. The combination of multimodal imaging with synergistic therapy (PTT/CDT/PDT) enhanced the efficacy of CB3@PEG in anticancer treatment [171]. Additionally, Li *et al.* constructed the multifunctional nanoplatform HA-ICG-Fe-PDA (Fig. 12C) for diagnosis and treatment of triple-negative breast cancer (TNBC). *In vitro* and *in vivo* experiments demonstrated that this platform possessed TNBC targeting, multimodal imaging and synergistic therapy capabilities, with good biocompatibility, providing new strategies for imaging-guided TNBC treatment [172]. Zhang constructed bismuth-core/iron-porphyrin covalent organic polymer-shell nanocomposites (BSCF) in 2024, which not only integrated PTT, PDT and CDT functions but also provided multimodal imaging capabilities [173].

Core-shell nanostructures are a common material type in synergistic therapy. Wang *et al.* designed the multifunctional nanoplatform SMP@I NPs (Figure 12D), with mesoporous SiO2 as the core, surface-coupled glucose oxidase (GOD) to produce H2O2, MnO2 shell to consume glutathione (GSH), polydopamine (PDA) layer for photothermal effects, and loaded ICG for PDT. Both *in vitro* and *in vivo* experiments showed that SMP@I NPs could effectively inhibit tumor growth, providing new strategies for cancer treatment [174]. Qi *et al.* designed a novel Fe3O4@Au/PPy-DOX nanoplatform to enhance cancer treatment efficacy [175]. Yan *et al.* used ZGGC as the core, coated with PDA, PEI, Si-Pc and adsorbed Cu2+, designing a nanoplatform ZPPSC based on persistent luminescence nanoparticles (PLNPs) for single-laser-activated synergistic PDT/PTT/CDT [176]. Wang introduced the MnO2/Ag3SbS3 (Fig. 12E) nanoplatform, achieving single-laser-triggered NIR-II PTT/PDT under 1064 nm laser irradiation, while degrading in acidic tumor microenvironment to produce •OH through Fenton-like reactions, catalyzing endogenous H2O2 to generate O2 and consume excess glutathione, alleviating tumor hypoxia and enhancing PDT/CDT effects, also suitable for tumor photoacoustic and magnetic resonance imaging [177]. Recently, more core-shell structured nanostructures, which can mediate PDT/PTT/CDT were designed and shown the good targeting, biocompatibility and stability [178,179,180].

**Table 3.** Multimodal photosensitive materials mediating the synergistic treatment of PDT, PPT and CDT.

Nanomaterials	PSs	Photothermic agent	catalyst	Materials	Ref.
**organic photosensitizers-Based Nanomaterials**	ICG	ICG	Ferrocene	ICG-MSN-Fe-CD	[161]
	ICG	ICG, PDA	Fe^3+^	HA-ICG-Fe-PDA	[172]
	ICG	PDA	Mn^2+^	SMP@I NPs	[174]
	ICG	Au_2_Pt	Au_2_Pt	APIJNM	[169]
	ICG	Co_3_S_4_-ICG	Co^2+^	Co_3_S_4_-ICG	[164]
	ICG	Pd_1.7_Bi@CeO_2、_ICG	Pd_1.7_Bi@CeO_2_	Pd_1.7_Bi@CeO_2_-ICG (PBCI)	[178]
	Ce6	Au_2_Pt	Au_2_Pt	Au_2_Pt-PEG-Ce6	[170]
	Ce6	Fe-TA	Fe^3+^	UCFS@Fe-TA	[168]
	TCPP	HMPB	Cu^2+^	HMPB@TCPP-Cu	[166]
	COP	Bi	Fe^2+^	BSCF	[173]
	IR780	IR780	hemin	BSA-Hemin-IR780 (dBHI)	[163]
	PCN-224	Pt	Cu^2+^	PCN-224/Pt/Cu^2+^ (FA-PPC)	[167]
**Inorganic photosensitizers-Based Nanomaterials**	Si-Pc	PDA	Cu^2+^	ZPPSC( ZGGC-PDA-PEI-Si-Pc-Cu)	[176]
	GCSR	GCSR	CuS	AuNBP@CuS@RBM(GCSR)	[179]
	Au_25_(NAMB)_18_ NCs	Au_25_(NAMB)_18_ NCs-Cu^2+^@SA-HA NHGs	Cu^2+^	Au_25_(NAMB)_18_ NCs-Cu^2+^@SA-HA NHGs	[180]
	Ag_3_SbS_3_	MnO_2_/Ag_3_SbS_3_(MA)	Mn^2+^	MnO_2_/Ag_3_SbS_3_(MA) NPs	[177]
	Fe_3_O_4_@Au/PPy	ppy and Au NPs	Fe_3_O_4_	Fe_3_O_4_@Au/PPy-DOX	[175]
	Bi_2_S_3_@Bi NRs	Bi_2_S_3_@Bi NRs	Art	Bi_2_S_3_@Bi@PDA-HA/Art NRs	[165]
	WO_3−x_@HA	WO_3−x_@HA	WO_3−x_@HA	WO_3−x_@HA	[162]
	Cu_2−x_Se/Bi_2_Se_3_	Cu_2−x_Se/Bi_2_Se_3_	Cu^2+^	Cu_2−x_Se/Bi_2_Se_3_@PEG	[171]

### Summary and Outlook

Currently, combining PDT with other therapeutic approaches like PTT, CDT, GT, ST, and PAT has made notable advancements in cancer treatment. By designing multifunctional nanoplatforms, researchers intended to overcome the limitations of traditional phototherapies, such as tumor microenvironment hypoxia and thermal resistance, to enhance therapeutic efficacy. These materials not only mediating PDT but also further amplify treatment effects by integrating PTT, CDT, GT, ST, and PAT. Additionally, many studies have incorporated multimodal imaging capabilities, such as photoacoustic imaging, magnetic resonance imaging (MRI), and computed tomography (CT), enabling precise tumor diagnosis and treatment.

Combination therapy, by integrating two or more different treatment methods, is able to more effectively kill tumor cells, increase the success rate of treatment and overcome the resistance of monotherapy. However, combination therapy faces challenges, such as the possibility that interactions between strategies may cause new or more serious side effects, higher management complexity and cost, and how to match the most suitable combination for the patient need further research. With continuous advancements in nanotechnology, the design of multifunctional nanoplatforms will become more intelligent, capable of responding to specific signals in the tumor microenvironment (such as pH, GSH, H2O2, etc.), enabling precise drug release and targeted therapy. Simultaneously, the integration of emerging therapeutic approaches like immunotherapy and gene therapy will allow phototherapy to play an even greater role in cancer treatment, especially in suppressing tumor recurrence and metastasis.

In summary, with the development of materials science, the synergistic application of PDT with other treatment modalities provides new perspectives and technical support for tumor treatment. In the future, multidisciplinary integration and technological innovation are expected to break through the bottlenecks of existing therapeutic strategies, offering cancer patients more efficient and safer treatment options.
